# Modulating the Molecular Geometry and Solution Self-Assembly of Amphiphilic Polypeptoid Block Copolymers by Side Chain Branching Pattern

**DOI:** 10.1021/jacs.1c01088

**Published:** 2021-04-06

**Authors:** Liying Kang, Albert Chao, Meng Zhang, Tianyi Yu, Jun Wang, Qi Wang, Huihui Yu, Naisheng Jiang, Donghui Zhang

**Affiliations:** † School of Materials Science and Engineering, University of Science and Technology Beijing, Beijing 100083, China; ‡ Department of Chemistry and Macromolecular Studies Group, Louisiana State University, Baton Rouge, Louisiana 70803, United States

## Abstract

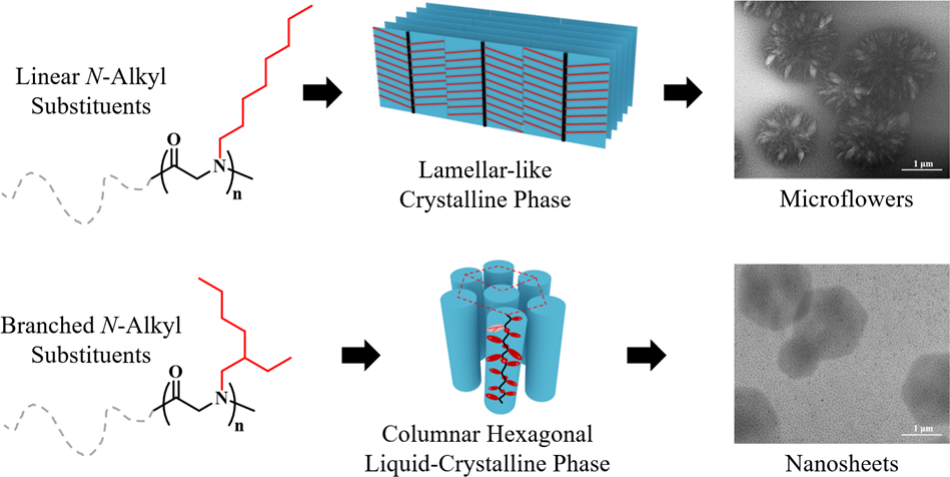

Solution self-assembly of coil-crystalline diblock copolypeptoids has attracted increasing attention due to its capability to form hierarchical nanostructures with tailorable morphologies and functionalities. While the N-substituent (or side chain) structures are known to affect the crystallization of polypeptoids, their roles in dictating the hierarchical solution self-assembly of diblock copolypeptoids are not fully understood. Herein, we designed and synthesized two types of diblock copolypeptoids, i.e., poly(*N*-methylglycine)-*b*-poly(*N*-octylglycine) (PNMG-*b*-PNOG) and poly(*N*-methylglycine)-*b*-poly(*N*-2-ethyl-1-hexylglycine) (PNMG-*b*-PNEHG), to investigate the influence of N-substituent structure on the crystalline packing and hierarchical self-assembly of diblock copolypeptoids in methanol. With a linear aliphatic N-substituent, the PNOG blocks pack into a highly ordered crystalline structure with a board-like molecular geometry, resulting in the self-assembly of PNMG-*b*-PNOG molecules into a hierarchical microflower morphology composed of radially arranged nanoribbon subunits. By contrast, the PNEHG blocks bearing bulky branched aliphatic N-substituents are rod-like and prefer to stack into a columnar hexagonal liquid crystalline mesophase, which drives PNMG-*b*-PNEHG molecules to self-assemble into symmetrical hexagonal nanosheets in solution. A combination of time-dependent small/wide-angle X-ray scattering and microscopic imaging analysis further revealed the self-assembly mechanisms for the formation of these microflowers and hexagonal nanosheets. These results highlight the significant impact of the N-substituent architecture (i.e., linear versus branched) on the supramolecular self-assembly of diblock copolypeptoids in solution, which can serve as an effective strategy to tune the geometry and hierarchical structure of polypeptoid-based nanomaterials.

## Introduction

Solution self-assembly of amphiphilic block copolymers that comprise at least one crystallizable blocks is an effective method for the generation of nonspherical polymeric micelles or nanoparticles with structural anisotropy and hierarchy. This is also known as the so-called crystallization-driven self-assembly (CDSA) process, where the self-assembly pathway and final morphology are mainly determined by the epitaxial crystalline growth of macromolecular building blocks in solution.^1,2^ Similar to CDSA, one can also utilize the molecular packing of liquid-crystalline-like molecules in solution to generate nonspherical nanoparticles, known as a liquid-crystallization-driven self-assembly (LCDSA) process.^3−5^ The access to well-defined one-dimensional (1D) nanofibers^6^ or nanorods,^7,8^ two-dimensional (2D) disks or nanosheets,^9,10^ or various multidimensional hierarchical self-assemblies^11^ via CDSA or LCDSA is of significant interest in the materials science community for their many emerging applications. In particular, nonspherical nanomaterials formed by the solution self-assembly of biocompatible macromolecules exhibit unique properties in many biomedical and biotechnological applications, such as drug/gene delivery^12,13^ and biomineralization.^14^ For example, relative to spherical nanoparticles, elongated filomicelles or nanodisks can either lead to longer blood circulation time^13^ or promote cell exterior binding with reduced cell uptake.^15^ From a fundamental standpoint, understanding how macromolecules self-assemble into well-defined anisotropic structures with hierarchy at micro/nanoscale also sheds new light on molecular biomimicry, as these structural features are ubiquitous in nature and can provide a wide variety of biological functions. For instance, cell membranes, a class of naturally occurring 2D structures, are selectively permeable to small molecules and can self-repair after damage.^16^

Many crystalline or liquid-crystalline polymers have been utilized as the primary core-forming building blocks to facilitate the CDSA or LCDSA of block copolymers in solution, including flexible linear polymers (e.g., polyethylene,^17,18^ poly(ε-caprolactone),^19^ poly(l-lactide))^20^ and polymers that have either relatively rigid backbones or bulky side groups (e.g., poly(2-(perfluorooctyl)ethyl methacrylate,^3,4^ poly(γ-benzyl-l-glutamate),^5^ polyferrocenylsilanes,^7,8,11,21^ and polythiophenes).^22,23^ In these case, the self-assembly pathway and final solution morphology are strongly dependent on chemical composition, block ratios, polymer solvent interactions, and inter/intramolecular interactions of the crystallizable or liquid crystalline blocks.^2,10,20,24,25^ In some cases, the solution self-assembly of crystallizable and liquid crystalline block copolymers can proceed in a living fashion, enabling access to low-dispersity anisotropic nanostructures or hierarchical assemblies with varying levels of structural complexity.^21,26,27^ It should be noted that for crystallizable and liquid crystalline polymers (e.g., polyfluorenes or polythiophenes),^28,29^ side chain engineering often serves as an effective strategy to modulate their inter- and intramolecular interactions and packing, thus allowing their morphology, solubility, and functionality to be systematically tailored. Yet, how the side chain geometry influences CDSA or LCDSA of block copolymers in solution remains largely unexplored.

Polypeptoids featuring N-substituted polyglycine backbones are structural mimics of polypeptides^30−32^ that possess excellent cytocompatibility and biodegradability.^33−35^ Owing to the absence of hydrogen-bonding and stereogenic centers along the N-substituted backbone, polypeptoids exhibit enhanced protease stability, good solubility, and thermal processability, in sharp contrast to polypeptides.^36,37^ Recent development of controlled polymerization methods also enabled the access to well-defined polypeptoids with diverse N-substituent structure and tunable molecular sequences,^34,38−45^ setting the stage for the systematic investigation of their crystallization and self-assembly behaviors. It is known that poly(*N*-methylglycine) (PNMG) (*a.k.a.* polysarcosine), i.e., the simplest member of the polypeptoid family, is amorphous in the solid state and can be readily dissolved in water or alcohol.^30,38,46,47^ By contrast, polypeptoids bearing relatively long linear *n*-alkyl side chains (4 ≤ *S* ≤ 14, where *S* is the number of carbon atoms in the linear *n*-alkyl group) are crystallizable and exhibit two phase transitions, i.e., a crystalline phase and a “sanidic” liquid crystalline (LC) mesophase, prior to isotropic melting.^40,43^ It should be noted that crystallization of these comb-shaped polypeptoids can occur at a relatively low number-average degree of polymerization (e.g., DP_n_ = 9).^42,43^ In the crystalline phase, the polypeptoid chains adopt a board-like structure where the backbone is fully extended in an all-*cis*-amide conformation and is approximately coplanar with the linear *n*-alkyl side chains (Figure S1).^43,48,49^ As a result, comb-shaped crystallizable polypeptoids often serve as useful candidates for the design and fabrication of polypeptoid-based anisotropic nanostructures or hierarchical assemblies in solution via CDSA. Sun and co-workers revealed the nanosheet formation of diblock copolymers composed of a hydrophilic coil-like poly(ethylene glycol) block and a hydrophobic crystalline poly(*N*-octylglycine) or poly(*N*-(2-phenylethyl)glycine) block in selective solvents.^45^ They also found that the formation of 2D nanosheets or planar brush-like nanostructures can occur by a hierarchical self-assembly process involving the initial formation of 1D nanofibrils.^44,50^ Our recent study has revealed the disparate self-assembly pathways of poly(*N*-methylglycine)-*b*-poly(*N*-decylglycine) diblock copolypeptoids with varying volume fractions of the solvophobic and crystallizable poly(*N*-decylglycine) block to form nanofibrils, nanorods, or nanosheets in methanol solution.^47^

When *n*-alkyl side chains are asymmetrically branched, e.g., in the case of *racemic* 2-ethyl-l-hexyl side chains, it was found that poly(*N*-2-ethyl-1-hexylglycine) (PNEHG) homopolymer (DP_n_ > 100) exhibits a single first-order thermal transition with a small enthalpic change.^40^ Below the thermal transition temperature, PNEHG polymers form a liquid crystalline mesophase with short-range ordering, evidenced by the presence of a single diffraction peak in the wide-angle X-ray scattering (WAXS) diffractogram.^40^ By contrast, for short PNEHG chains (DP_n_ ≤ 20), no first-order transition was observed within a broad temperature range by differential scanning calorimetry (DSC), indicating PNEHG homopolymers or block copolymers with relatively low DP_n_ are amorphous.^51,52^ While these previous findings clearly indicate that the molecular packing of polypeptoids can be significantly altered by the presence of branched aliphatic side chains relative to those bearing linear side chains, how the branching pattern of the N-substituent influences the molecular packing and the supramolecular self-assembly of their block copolymers in solution remains unclear.

In this contribution, we designed and synthesized two types of diblock copolypeptoids that are composed of a core-forming block with either linear or branched *n*-alkyl side chains, namely, poly(*N*-methylglycine)-*b*-poly(*N*-octylglycine) (PNMG-*b*-PNOG) and poly(*N*-methylglycine)-*b*-poly(*N*-2-ethyl-1-hexylglycine) (PNMG-*b*-PNEHG), with nearly identical molecular weight (*M*_n_) and block composition by a controlled ring-opening polymerization (ROP) method. The solution self-assembly of these two types of diblock copolypeptoids in methanol was further investigated by small-/wide-angle X-ray scattering (SAXS/WAXS) in conjunction with cryogenic transmission electron microscopy (cryo-TEM) and atomic force microscopy (AFM) techniques to probe the effect of side chain architecture on supramolecular self-assembly. We show that PNMG-*b*-PNOG bearing linear *n*-octyl side chains slowly self-assembled into hierarchical flower-like aggregates composed of radially distributed nanoribbons, driven by the crystalline packing of board-like PNOG blocks. By contrast, PNMG-*b*-PNEHG bearing branched *racemic* 2-ethyl-1-hexyl side chains self-assembles into well-defined hexagonal nanosheets, resulting from the LC packing of rod-shaped PNEHG in a hexagonal lattice in 2D, i.e., a columnar hexagonal LC mesophase. These results highlight the significant role of side chain architecture on the molecular packing and hierarchical self-assembly of diblock copolypeptoids, which can be used to tune the shape, anisotropy, and hierarchical complexity of polypeptoid-based self-assemblies in solution.

## Results and Discussion

### Polymer Synthesis and Solution Preparation

**Scheme 1:**
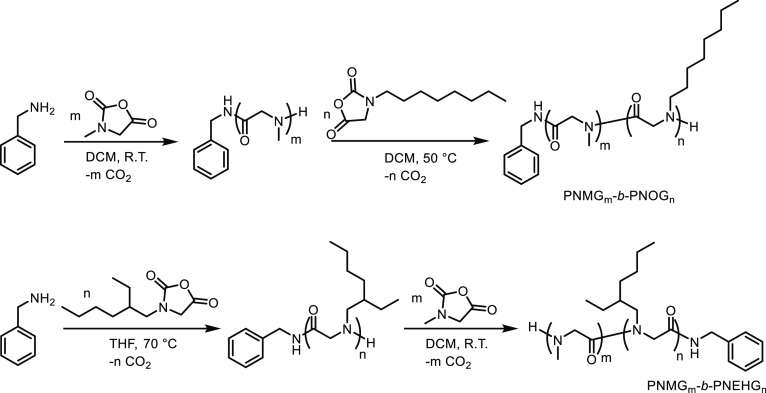


Poly(*N*-methylglycine)-*b*-poly(*N*-octylglycine) (PNMG-*b*-PNOG) and poly(*N*-methylglycine)-*b*-poly(*N*-2-ethyl-1-hexylglycine) (PNMG-*b*-PNEHG) diblock copolypeptoids with comparable molecular weight and block composition were synthesized by benzylamine-initiated ring-opening polymerization of the corresponding N-substituted *N*-carboxyanhydrides (R-NCAs) in a sequential manner (Scheme 1-2). The detailed synthesis protocol is summarized in the Experimental Section (see the Supporting Information (SI)). In brief, sequential polymerizations of *N*-methyl *N*-carboxyanhydride (Me-NCA) (M_1_) and *N*-octyl *N*-carboxyanhydride (Oct-NCA) (M_2_) were performed in DCM (a good solvent for both monomers and the final polymer) using a benzylamine initiator to generate PNMG-*b*-PNOG in a one-pot fashion. Due to solubility issues, the synthesis of PNMG-*b*-PNEHG was performed in two steps involving a change of solvent. *N*-2-Ethyl-1-hexyl *N*-carboxyanhydride (EtHex-NCA) was first polymerized in THF using benzylamine initiator to produce a PNEHG segment. The polymer was then dried and redissolved in DCM, to which Me-NCA monomers were subsequently introduced and polymerized to produce the PNMG-*b*-PNEHG block copolymer. The experimental compositions of PNMG-*b*-PNOG and PNMG-*b*-PNEHG were determined by the end-group analysis using ^1^H NMR spectra (Figures S2–S5) and are summarized in Table 1. The polydispersity index (PDI) of the polymers (Figure S6) was determined by size-exclusion chromatography (SEC) coupled with a differential refractive index (dRI) detector using poly(methyl methacrylate) (PMMA) standards in 1,1,1,3,3,3-hexafluoro-2-propanol (HFIP) with CF_3_CO_2_K (0.05 M). The initial monomer to initiator ratios, i.e., [M_1_]_0_:[M_2_]_0_:[I]_0_, were kept constant at 100:100:1 to ensure that two different polymers have similar block composition, i.e., the chain length of the solvophobic PNOG or PNEHG block and that of the solvophilic PNMG block. As the PNMG block is solvophilic and the PNOG or PNEHG block is relatively solvophobic,^38,40,47^ both PNMG-*b*-PNOG and PNMG-*b*-PNEHG block copolymers are expected to form core–shell-type aggregates in methanol. We have found that both diblock copolypeptoids can be readily dissolved at a 5 mg/mL concentration in methanol with heating at ∼80 °C (for PNMG-*b*-PNOG) or ∼100 °C (for PNMG-*b*-PNEHG) for 10 min in sealed glass vials, producing visually clear solutions. These temperatures are sufficient to induce the melting of PNOG and PNEHG blocks in methanol, evidenced by high-temperature SAXS/WAXS (*vide infra*). CDSA or LCDSA of PNMG-*b*-PNOG and PNMG-*b*-PNEHG was then triggered by cooling of the respective methanol solution from high temperature to room temperature within 10 min. The average cooling rate is approximately 6–8 °C/min. The solutions were kept at room temperature in sealed glass vials for at least 1 day prior to further structural characterization by microscopy and X-ray scattering methods. Note that such time is sufficient for the completion of the self-assembly process for both polymers in a 5 mg/mL methanol solution (*vide infra*). Aside from solution scattering and cryo-TEM, we also confirmed that the structural characterizations using other *ex situ* techniques, such as AFM and regular TEM, which involve either drop-coating or spin-coating during sample preparation, are experimentally valid in revealing the solution morphology of PNMG-*b*-PNOG and PNMG-*b*-PNEHG assemblies (Figure S7).

**Table 1:** Molecular Characteristics of PNMG-*b*-PNOG and PNMG-*b*-PNEHG Polymers

sample	[M_1_]_0_:[M_2_]_0_:[I]_0_^a^	actual polymer composition^b^	*M*_n_ (theor) (kg/mol)^c^	*M*_n_ (NMR) (kg/mol)^d^	*M*_n_ (SEC) (kg/mol)^e^	PDI^e^	*f*_PNOG_ or *f*_PNEHG_^f^
PNMG-*b*-PNOG	100:100:1	PNMG_116_-*b*-PNOG_94_	24.1	24.2	23.0	1.15	0.73
PNMG-*b*-PNEHG	100:100:1	PNMG_121_-*b*-PNEHG_101_	24.1	25.8	21.5	1.18	0.74

a Initial monomer-to-initiator ratio, where M_1_ corresponds to Me-NCA, and M_2_ corresponds to Oct-NCA or EtHex-NCA.

b The numbers in subscripts correspond to the actual DP_n_ of an individual block determined by end-group analysis using ^1^H NMR spectroscopy in CD_2_Cl_2_.

c Theoretical molecular weights were calculated from the initial monomer-to-initiator ratio.

d Determined by ^1^H NMR analysis.

e Determined by the SEC-dRI method using PMMA standards (HFIP/CF_3_CO_2_K (0.05 M), 30 °C).

f The volume fraction of PNOG (*f*_PNOG_) or PNEHG block (*f*_PNEHG_) was calculated from the block copolymer composition and the density for each block.

### Solution Self-Assembly of PNMG-*b*-PNOG Diblock Copolypeptoids Bearing Linear Aliphatic Side Chains

**Figure 1:**
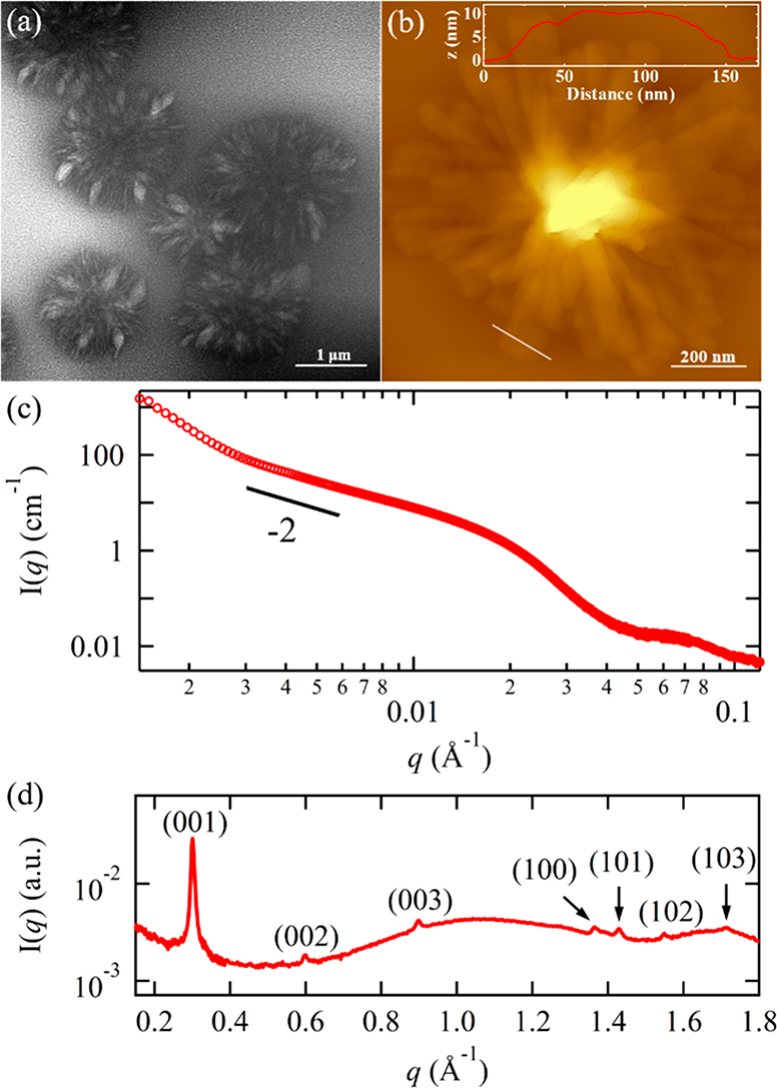
(a) Cryo-TEM and (b) AFM images for PNMG-*b*-PNOG in diluted methanol solution. The inset of (b) shows the height profile (from the Si surface) along the white line obtained from the AFM cross-sectional analysis. (c) SAXS and (d) WAXS intensity profiles for the 5 mg/mL PNMG-*b*-PNOG in methanol.

Cryo-TEM imaging of the vitrified PNMG-*b*-PNOG methanol solution (0.5 mg/mL obtained by dilution from a 5 mg/mL solution after self-assembly) has revealed the formation of hierarchical flower-like assemblies (i.e., microflowers) with an average diameter of ∼1.1 μm (Figure 1a). Similar structures were also found by AFM (Figure 1b) and regular TEM analysis (Figure S7a) after depositing the same solution onto silicon substrates or carbon grids. A closer inspection reveals that these microflowers are composed of multiple elongated 2D nanoribbons that stacked radially into three dimensions (Figure 1b) (Figure S7a). While the length and width of the nanoribbons are somewhat variable (ca. 500 ± 50 nm and 120 ± 30 nm, respectively), the thickness of the nanoribbon is consistently measured to be 11 ± 1 nm by AFM analysis. Note that different initial concentrations of the PNMG-*b*-PNOG solutions in the dilute regime (0.05–5 mg/mL) all afforded a microflower morphology at the final stage of self-assembly (Figure S8).

To obtain detailed structural information regarding the molecular arrangement inside these microflowers, we conducted small/wide-angle X-ray scattering experiments on the 5 mg/mL self-assembled PNMG-*b*-PNOG methanol solution using a capillary flow cell at 20 °C. As shown in Figure 1c, the scattering intensity exhibits a power law dependence on *q* with a −2 exponent at the intermediate *q* range, before giving way to a broad shoulder at *q* ≈ 0.02 Å^–1^, suggesting the formation of 2D nanostructures (i.e., the nanoribbons) with a lateral size of a few tens to hundreds of nanometers. A scattering peak near *q* ≈ 0.07 Å^–1^ is also discernible, which is likely to be associated with the geometric cross-section of the nanoribbons based on AFM results (Figure 1b). From the intensity minimum at *q* ≈ 0.045 Å^–1^, the thickness of the nanoribbons is estimated to be ∼14 nm by the *d* = 2π/*q* relationship. In addition, a *I*(*q*) ∼ *q*^–4^ behavior is observed at the lowest *q* region (i.e., *q* < 0.003 Å^–1^), indicating the presence of sharp interfaces, which are attributed to the interfaces between polymeric aggregates and solvent. These scattering features are consistent with the hierarchical microflowers revealed by cryo-TEM and AFM imaging analysis. In the WAXS region (Figure 1d), a sharp scattering peak was observed at *q* = 0.30 Å^–1^, which corresponds to the distance between adjacent backbones of PNOG segments that are separated by the linear *n*-octyl side chains in the crystalline lattice,^40,44,47,48^ i.e., the (001) packing along the crystallographic *c*-axis, as illustrated in Figure S1 and Figure 6. The presence of higher order (002) and (003) peaks together with the (100), (101), and (102) peaks provides strong evidence for the crystalline packing of the core-forming PNOG blocks adopting a board-like molecular geometry where the backbones are fully extended in an all-*cis*-amide conformation and nearly coplanar with the *N*-alkyl side chains splaying on either side of the backbone.^40,47,48^

**Figure 2:**
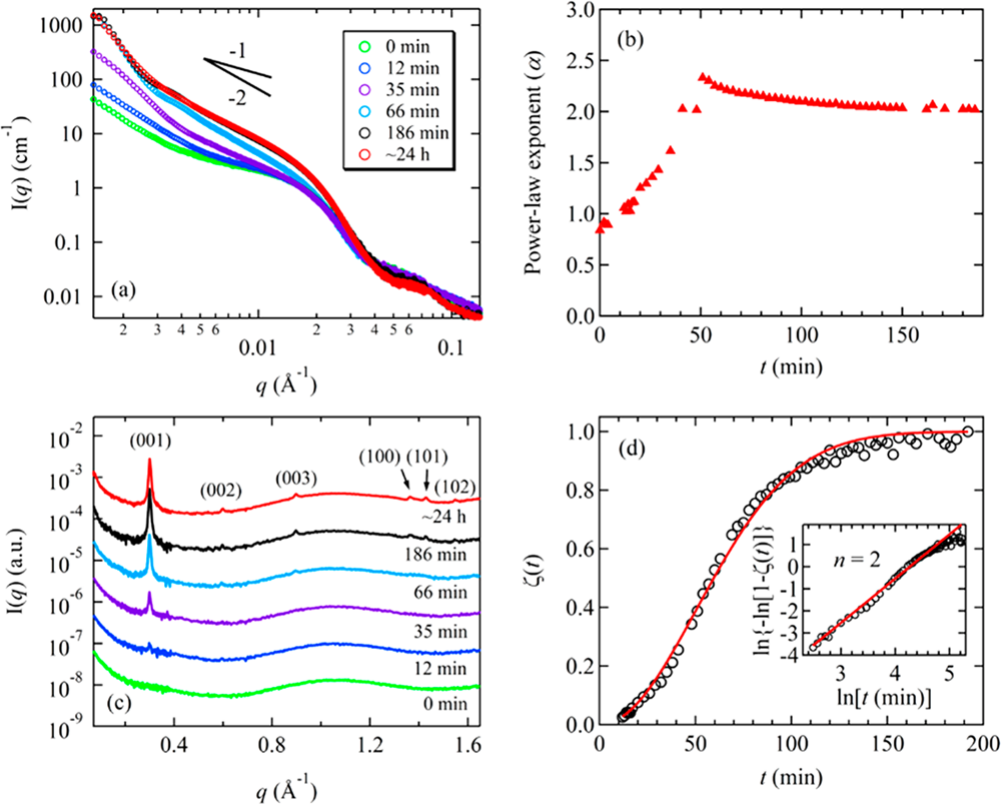
(a) Representative SAXS profiles of the 5 mg/mL PNMG-*b*-PNOG methanol solution at different waiting times (*t*) after being cooled to room temperature. (b) Plot of the exponent (*a*) values of *I*(*q*) ∼ *q*^–*a*^ near *q* = 0.006 Å^–1^ as a function of *t*. (c) Corresponding WAXS profiles at different *t*, where the data have been shifted vertically for clarity. (d) ζ(*t*) values (black circles) obtained from the normalized integrated intensity of the (001) peak at different *t*. Inset of (d) shows the corresponding Sharp–Hancock plot. The red solid lines in (d) correspond to the best fits to the data using eq 1 with *n* = 2.

**Figure 3:**
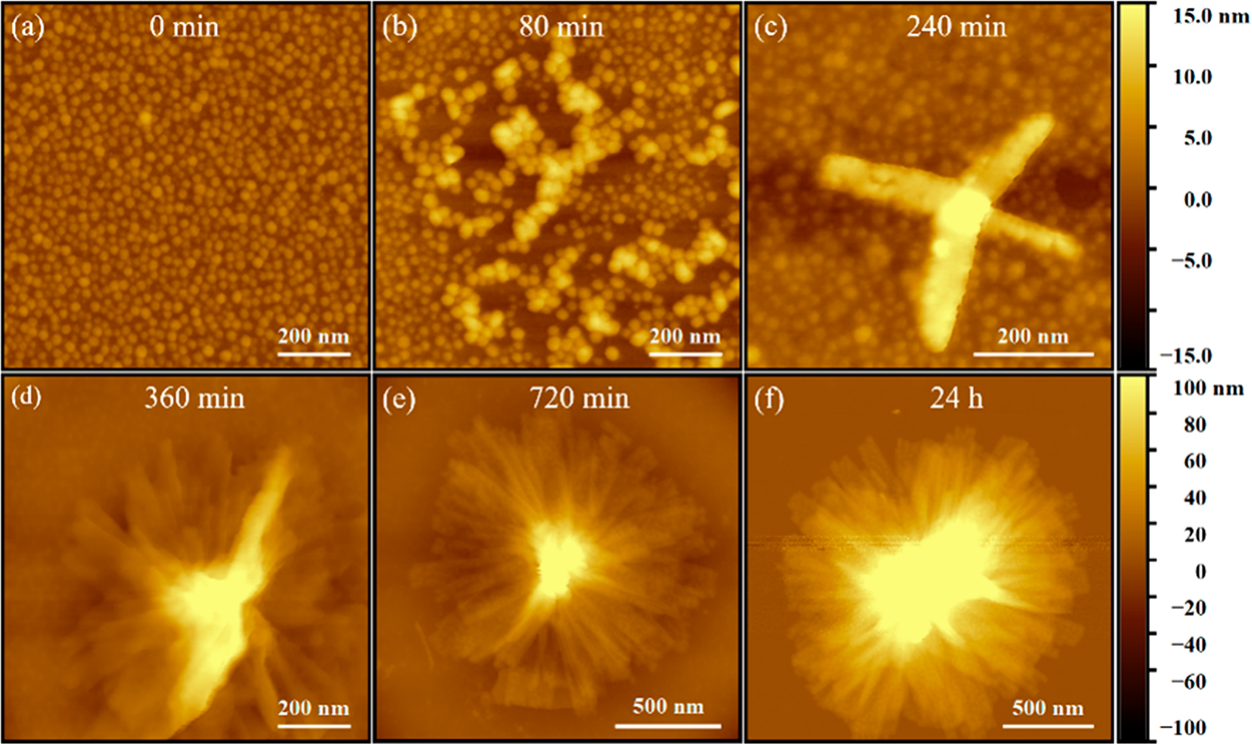
AFM height images for the PNMG-*b*-PNOG self-assemblies at different waiting times (*t*) after the initial 0.5 mg/mL solution had been cooled to room temperature: (a) 0 min, (b) 80 min, (c) 240 min, (d) 360 min, (e) 720 min, and (f) 24 h. Note that the 0.5 mg/mL solution was immediately deposited onto Si substrates at each given time interval. The height scales for the top row (a–c) and bottom row (d–f) are ±15 and ±100 nm, respectively.

**Figure 4:**
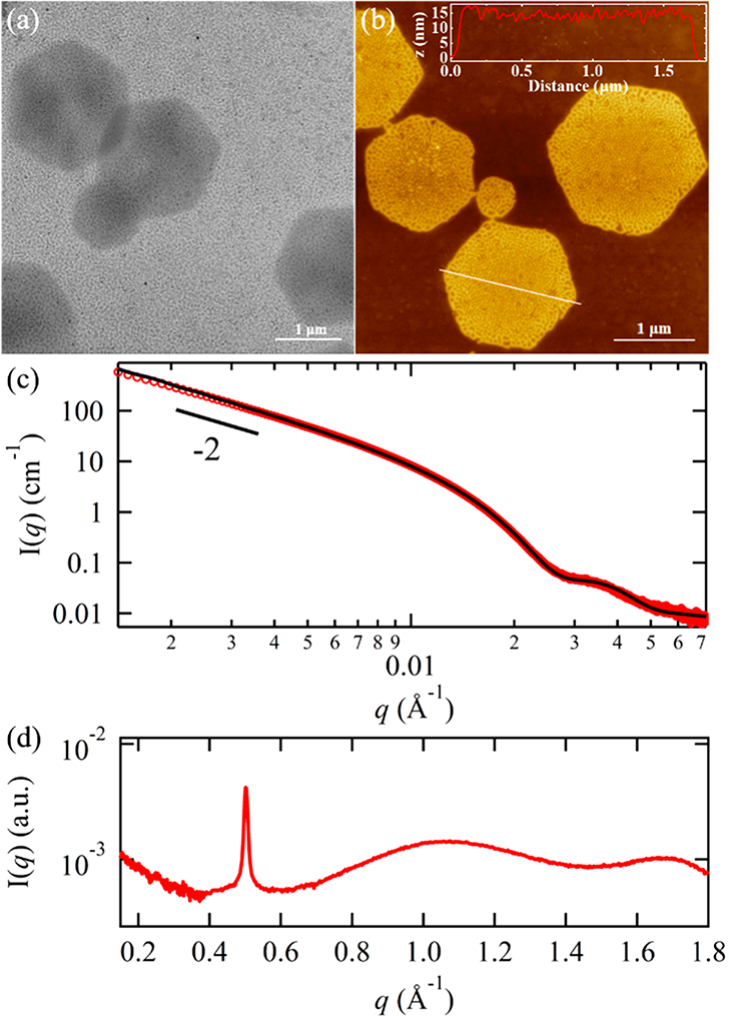
Cryo-TEM (a) and AFM (b) images for PNMG-*b*-PNEHG in a diluted methanol solution (1 mg/mL). The inset of (b) shows the height profiles (from the Si surface) along the white line obtained from the AFM cross-sectional analysis. (c) SAXS and (d) WAXS intensity profile for the 5 mg/mL PNMG-*b*-PNEHG in methanol. The best-fit line to the SAXS data of the PNMG-*b*-PNEHG solution is based on the scattering model described in the text.

**Figure 5:**
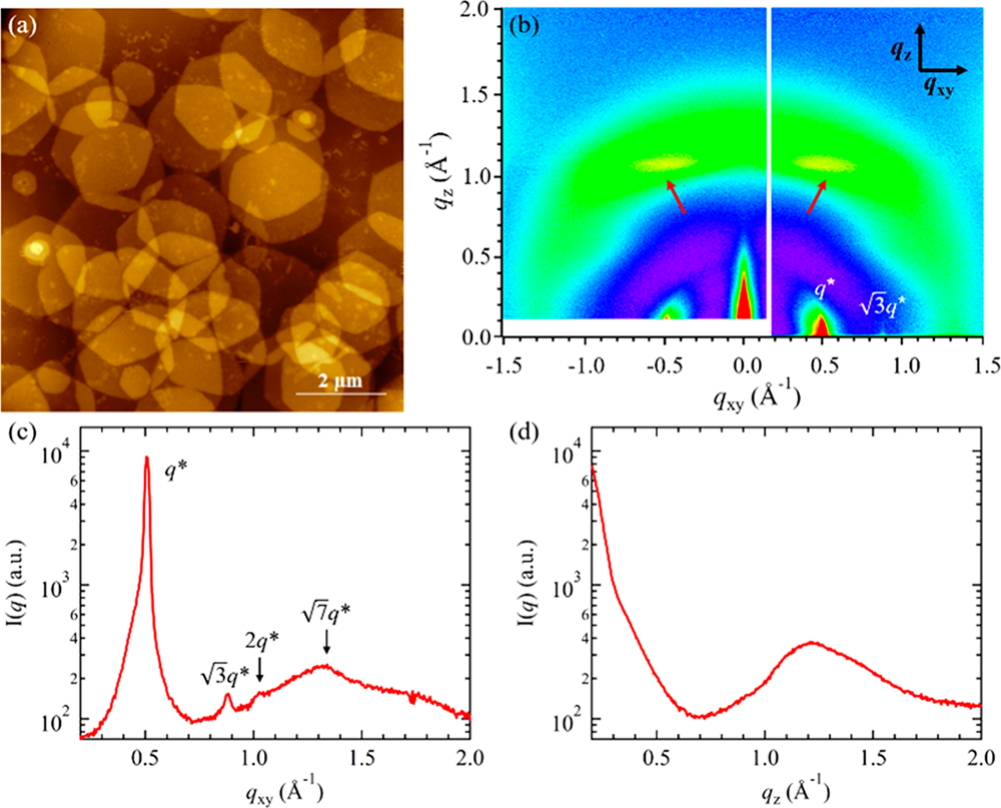
(a) AFM image and (b) 2D GIWAXD image for the PNMG-*b*-PNEHG hexagonal nanosheets deposited onto a Si substrate. The two off-axis streaks are indicated by red arrows in (b). The corresponding one-dimensional GIWAXD profiles along the *q*_*xy*_ direction and *q*_*z*_ direction are shown in (c) and (d), respectively.

**Figure 6:**
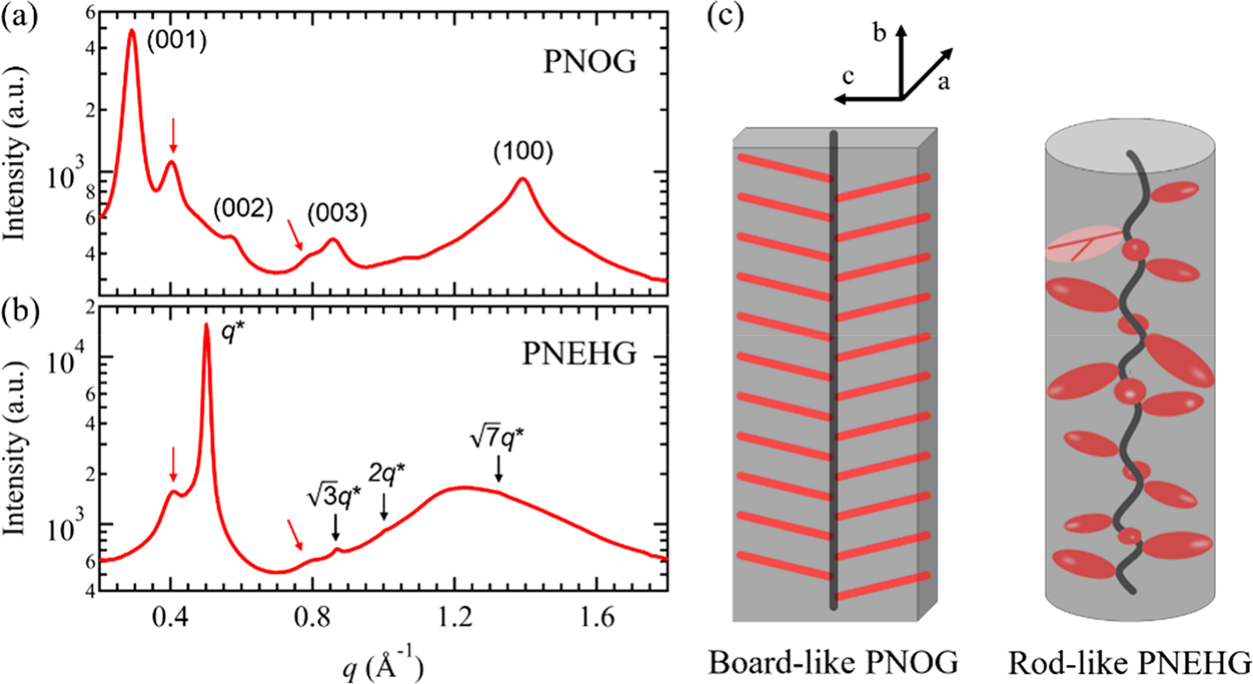
One-dimensional WAXD profiles of the bulk (a) PNOG and (b) PNEHG homopolymers after recrystallizing from the melt. The primary and secondary peaks associated with the Kapton windows on the sample cell are indicated by the red arrows. (c) Proposed molecular geometries of PNOG and PNEHG at the crystalline/liquid crystalline state.

*In situ* SAXS analysis of the methanol solution of PNMG-*b*-PNOG polymers (5 mg/mL) at 70 °C revealed significantly reduced scattering intensity in the entire *q* range relative to that at 20 °C (Figure S9a). The model-independent Guinier plot analysis of the SAXS data yielded a radius of gyration (*R*_g_) of 3.2 nm, which is consistent with that expected of PNMG-*b*-PNOG unimers. The slight low-*q* upturn in the SAXS profile suggests a low level of intermolecular aggregation in the solution. In addition, no diffraction peaks were observed in the WAXS profile of the methanol solution of PNMG-*b*-PNOG polymers (5 mg/mL) at 70 °C, in contrast to that at 20 °C (Figure S9b). These combined results indicate that most PNMG-*b*-PNOG molecules at 5 mg/mL concentration in methanol are dissociated by high temperature (70 °C) along with residual intermolecular aggregates in the solution. Crystallization and hierarchical self-assembly of the PNMG-*b*-PNOG molecules were triggered after cooling the solution to room temperature.

Immediately after the 5 mg/mL solution was supercooled to room temperature, the structural evolution of PNMG-*b*-PNOG as a function of time was monitored by SAXS/WAXS. As shown in Figure 2a,b, we observed a drastic change of the SAXS profile as a function of the waiting time (*t*). At the intermediate *q* region (i.e., near *q* = 0.006 Å^–1^), the dependence of *I*(*q*) over *q* changes from *I*(*q*) ∼ *q*^–0.8^ to *q*^–2.4^ during the early stage of self-assembly (*t* < 50 min), then gradually stabilizes at *I*(*q*) ∼ *q*^–2^ after ∼60 min. This indicates the occurrence of complex structural evolution toward a final solution morphology. The *I*(*q*) ∼ *q*^–2^ relationship at the intermediate *q* range at the final stage of self-assembly indicates the formation of 2D nanostructures with a few tens to hundreds of nanometers in average size, consistent with the nanoribbon subunits observed by cryo-TEM and AFM. The broad peak near 0.07 Å^–1^ becomes prominent after ∼66 min (Figure 2a and Figure S10), suggesting the formation of a well-defined cross-section of the nanosheets. Meanwhile, multiple peaks attributed to the crystalline packing of PNOG segments along the crystallographic *c*- and *a*-axes, i.e., the (001), (100), and higher order reflections, begin to appear after ∼12 min and intensify over time in the WAXS region (Figure 2c). The simultaneous evolution in both SAXS and WAXS profiles suggests a strong correlation between the hierarchical self-assembly of PNMG-*b*-PNOG and the crystallization of the core-forming PNOG blocks. To better understand the crystalline growth of PNOG, the time-dependent WAXS data were further analyzed by the Avrami–Erofeev expression:^53,54^
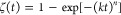
1where ζ(*t*) is the extent of crystallization (or relative crystallinity) at isothermal crystallization time *t*, *k* is the crystallization rate constant, and *n* is the Avrami exponent that depends on the growth geometry of the crystals (e.g., a value of 3 corresponds to unrestricted three-dimensional crystal growth and a value of 2 to two-dimensional crystal growth) as well as the time dependence of nucleation (e.g., 0 corresponds to instantaneous, athermal nucleation and 1 to purely sporadic, thermal nucleation).^55^Figure 2d shows the evolution of ζ(*t*) for the crystallization of PNOG blocks at room temperature, where ζ(*t*) was deduced from the integrated intensity of the (001) peak at a given time (*t*) normalized by its maximum value (i.e., the integrated intensity of the (001) peak at 24 h). The best fit to the ζ(*t*) profile using the Avrami–Erofeev equation (eq 1) gives *n* = 2 and *k* = 0.014 min^–1^, which can also be presented in the Sharp–Hancock form via ln{−ln[1 – ζ(*t*)]} = *n* ln *t* + *n* ln *k* (the inset of Figure 2d).^56^ The measured Avrami exponent of *n* = 2 is consistent with a two-dimensional crystallization of the PNOG blocks by an instantaneous, athermal nucleation process, resulting in the formation of the 2D nanoribbon subunits that constitute the PNMG-*b*-PNOG microflowers.^55,57^

The morphological evolution of PNMG-*b*-PNOG was further characterized using AFM by depositing the self-assembled nanostructures on Si substrates at a given time (Figure 3). Note that the initial solution concentration used for time-dependent AFM study is 0.5 mg/mL, which is 10 times diluted relative to the samples used for the time-dependent SAXS/WAXS study. At this concentration, the self-assembly of PNMG-*b*-PNOG is significantly retarded, which allows for the morphological change in a wide time window to be captured. At *t* = 0 min, where the solution was immediately deposited onto a Si substrate after cooling to room temperature, we observed well-dispersed spherical nanoparticles with an average diameter of ∼30 nm throughout the sample. The diameter is in good agreement with that determined (∼29.6 nm) by the analysis of the SAXS scattering profile at *t* = 0 min (Figure S11). Considering that the *in situ* high-temperature SAXS/WAXS measurements show the predominant presence of unimers at 70 °C (Figure S9a), these results clearly indicate the early association of PNMG-*b*-PNOG has occurred as the solution was cooled to room temperature, prior to the onset of crystallization. As shown in Figure 3, the coexistence of small spherical micelles and nanoribbons/large microflowers is observed during the intermediate stage of self-assembly (240 ≤ *t* ≤ 720 min). Driven by the crystallization of PNOG blocks, these early micelles serve as the primary building blocks for the subsequent self-assembly, promoting the growth of microflowers, until all available polymeric materials are consumed. We postulate that the 2D growth of nanoribbons and the formation of microflowers mainly involve crystalline-driven fusion and reorganization of the initially formed amorphous spherical micelles, similar to previous reports of nanorod and nanosheeting forming coil-comb-shaped polypeptoid diblock copolymers.^45,47^

### Solution Self-Assembly of PNMG-*b*-PNEHG Diblock Copolypeptoids Bearing Branched Side Chains

In contrast to the PNMG-*b*-PNOG block copolymer, which forms microflowers in methanol, PNMG-*b*-PNEHG molecules were found to self-assemble into symmetric 2D hexagonal nanosheets after the methanol solution was cooled to room temperature (Figure 4a,b and Figure S7b). While the lateral diameter of these hexagons varies significantly from hundreds of nanometers to a few micrometers, the thickness of hexagons is highly uniform at 16 ± 1 nm in the dry state, as determined by AFM analysis (Figure 4b). The SAXS analysis of the polymer aggregate solution exhibits a *I*(*q*) ∼ *q*^–2^ dependence in the 0.001–0.01 Å^–1^ low-*q* range (Figure 4c), consistent with the formation of 2D nanostructure. Apart from the broad shoulder at *q* ∼ 0.015 Å^–1^, the peak attributed to the thickness of hexagonal nanosheets is also discernible near *q* = 0.03 Å^–1^. Meanwhile, the WAXS profile (Figure 4d) of PNMG-*b*-PNEHG solution shows a single diffraction peak at *q* = 0.50 Å^–1^, which gives a *d*-spacing of 1.26 nm, corresponding to the distance between adjacent PNEHG segments that are separated by the interdigitated *N*-2-ethyl-1-hexyl side chains.^40^ Aside from the primary peak, we did not observe any higher order diffraction peaks, indicating the PNEHG segments form a liquid crystalline mesophase that lacks long-range molecular ordering. The liquid crystalline nature of PNEHG domains was also evidenced by the small enthalpic change associated with the corresponding thermal transition observed in the DSC thermogram of PNMG-*b*-PNEHG polymers (Figure S16).

Since the PNMG block is solvophilic and the PNEHG block is relatively solvophobic in methanol,^38,40,47^ the PNMG-*b*-PNEHG molecules are expected to form core–shell-type aggregates. Given the observed aggregates have a hexagonal sheet-like geometry, it is reasonable that the liquid-crystalline PNEHG blocks form a 2D hexagonal core, whereas the soluble PNMG blocks form a diffused corona surrounding the LC core to stabilize the hexagonal nanosheets in solution. The scattering form factor for core–corona disk-shaped micelles (*a.k.a.* block copolymer micelles with a disk-shaped core) developed by Pedersen and co-workers was applied to fit the SAXS profile of the 5 mg/mL PNMG-*b*-PNEHG methanol solution.^58,59^ The detailed description of the scattering model is summarized in the SI. From the best fit to the data, the core thickness (*H*_c_) was estimated to be 11.3 ± 0.2 nm with a polydispersity (σ_Hc_) of 0.2 (Figure S12). The radius of gyration of the corona chains (*R*_g,chain_) was estimated to be 2.7 ± 0.2 nm, which gives the value of the corona thickness (*h*_corona_) of 5.9 ± 0.4 nm by the geometrical relationship of *h*_corona_ = (*d*_int_ + 1.291)*R*_g,chain_, where *d*_int_ is close to unity to indicate nonpenetration of the corona chains into the core region (see the SI for details). Thus, the total thickness of the PNMG-*b*-PNEHG hexagonal nanosheets in methanol solution was estimated to be 23.1 ± 1.0 nm. The thickness determined by SAXS analysis is greater than that obtained by AFM analysis (16 ± 1 nm), which is attributed to the collapse of corona-forming PNMG chains in the AFM samples after solvent evaporation.

**Figure 7:**
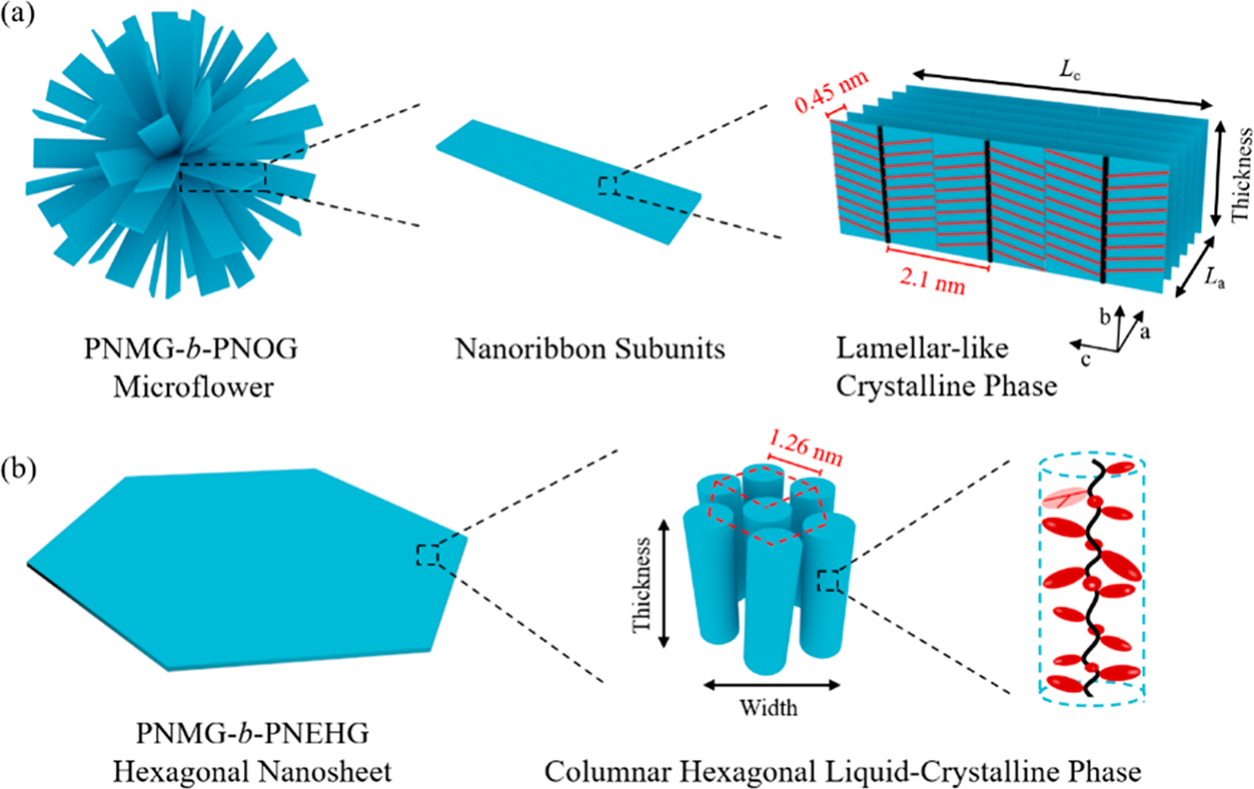
Schematic illustration of the molecular arrangement inside the PNMG-*b*-PNOG microflower and PNMG-*b*-PNEHG hexagonal nanosheet. The corona-forming PNMG blocks and possible chain folding of PNOG were omitted for clarity.

To further probe the molecular packing and orientation inside of the nanosheets, the self-assembled 5 mg/mL PNMG-*b*-PNEHG solution was deposited onto a Si substrate by spin-coating and subjected to grazing-incidence wide-angle X-ray diffraction (GIWAXD) measurements. As shown in Figure 5a, the large 2D hexagonal nanosheets were laid flat on the Si substrate due to geometric confinement. This preferential orientation allows the molecular orientation within the dried hexagonal nanosheets to be resolved using GIWAXD. Interestingly, aside from the primary diffraction peak at q* = 0.50 Å^–1^, multiple higher order peaks located at √3q*, √4q*, and √7q* along the in-plane (*q*_*xy*_) direction were also observed by GIWAXD (Figure 5b,c), indicating the liquid-crystalline PNEHG molecules are rod-like and packed into a hexagonal lattice with the long axis of the rods aligned normal to the substrate (and the surface of hexagonal nanosheets). The existence of such a columnar hexagonal phase of a mesogenic rod-like PNEHG homopolymer in the solid state is also confirmed by WAXD (*vide infra*). Hence, we conclude that the lateral dimension of the PNMG-*b*-PNEHG nanosheet is governed by the LC packing of rod-like PNEHG molecules in a 2D hexagonal lattice, whereas the thickness of the nanosheet core is determined by the height of hexagonal columns, as illustrated in Figure 7b. It should be noted that from the solution-state X-ray scattering results (Figure 4d) only the primary peak at q* = 0.50 Å^–1^ is observed. Nevertheless, the formation of symmetrical hexagonal nanosheets suggests that a columnar hexagonal phase of rod-like PNEHG molecules was formed within the core region of the sheet in the solution. As the higher order peaks associated with the columnar hexagonal lattice are relatively weak even in dry states (Figure 5) and bulk samples (see below, Figure 6), we postulate that these higher order peaks are possibly overwhelmed by the incoherent scattering from solution WAXS measurements using a capillary cell.

Aside from the strong reflections along *q*_*xy*_, we also observed two discrete off-axis streaks with low intensity that are aligned parallel to *q*_*xy*_, as indicated by the red arrows in Figure 5b. This scattering feature is reminiscent of a typical diffraction pattern for helical structures that exhibit off-meridional layer lines perpendicular to the helical axis (*a.k.a.* the characteristic X-pattern).^60^ Given the PNEHG segments are composed of a random distribution of *racemic N*-2-ethyl-1-hexyl side chains and no higher order off-meridional streaks were observed in the GIWAXD spectrum, the weak off-axis streaks in Figure 5b may suggest the presence of short and noncontinuous helix-like segments along the backbone in low abundance. A broad ring-like scattering with low intensity near *q* = 1.2 Å^–1^ (*d* = 5.2 Å) is also discernible, which is tentatively attributed to the amorphous regions of the polymer backbone.^40^ Based on the above scattering results, it is concluded that the PNEHG molecules adopted an extended backbone conformation with the bulky *N*-2-ethyl-1-hexyl side chains radially and outwardly displayed along the backbone, as illustrated in Figure 7b. This is presumably a consequence of minimizing the steric hindrance of the bulky side chains. We postulate that the formation of the PNMG-*b*-PNEHG hexagonal nanosheets is driven by the molecular packing of rod-like PNEHG segments with an extended chain conformation into a columnar hexagonal mesophase in the micellar core.

When the PNMG-*b*-PNEHG solution was heated to 100 °C, *in situ* high-temperature SAXS shows a relatively weak scattering (Figure S13a), which gives an *R*_g_ of 3.7 nm based on the Guinier plot analysis. Note that a certain degree of intermolecular aggregation was also evidenced by the sharp low-*q* upturn at *q* < 0.03 Å^–1^ in the SAXS profile (Figure S13a). Meanwhile, the diffraction peak at *q* = 0.50 Å^–1^ has largely disappeared in the WAXS region at 100 °C, indicating the formation of an isotropic disordered phase. By visual inspection, it was found that the 5 mg/mL PNMG-*b*-PNEHG solution became cloudy immediately after cooling below ∼90 °C. Drastic changes in the SAXS/WAXS profiles were also observed upon cooling the solution to room temperature (Figure S13b). In contrast to PNMG-*b*-PNOG molecules whose solution assembly occurs over the course of several hours, the LCDSA process of PNMG-*b*-PNEHG molecules to form hexagonal nanosheets occurred rapidly and reached completion immediately upon cooling below the clearing temperature (at which a transition from isotropic liquid phase to LC mesophase occurs). As shown in Figure S14, no change in the SAXS/WAXS profiles as a function of waiting time was observed after the solution was cooled to room temperature. This indicates that the solution self-assembly of PNMG-*b*-PNEHG molecules is much faster than that of PNMG-*b*-PNOG molecules at room temperature, which is likely due to the reduced structural ordering of the liquid crystalline phase relative to the crystalline phase formed in the aggregate core upon self-assembly for the former than the latter.

### Effect of Side Chain Branching on the Molecular Packing and Hierarchical Assembly of Diblock Copolypeptoids in Solution

From the above results, it is evident that the side chain branching has a profound impact on the molecular packing of the core-forming polypeptoid block, giving rise to different solution morphologies of the diblock copolypeptoids by disparate hierarchical assembly pathways. With linear aliphatic side chains, the PNOG blocks adopted the typical board-like conformation with a fully extended *cis*-amide backbone being coplanar with the *n*-octyl side chains in the crystalline lattice when cooled below *T*_c_. By contrast, the PNEHG blocks bearing *racemic* 2-ethyl-1-hexyl side chains are rod-like and self-assemble into a columnar hexagonal LC mesophase after being cooled from high temperature in solution, where the molecular ordering in the resulting aggregate was more short-ranged relative to that of the PNOGs. Consequently, the PNMG-*b*-PNOG molecules slowly self-assemble into microflowers composed of stacked nanoribbons in methanol, while the PNMG-*b*-PNEHG counterparts form hexagonal nanosheets at a much faster rate of self-assembly.

To better understand the nature of molecular packing of these core-forming blocks, we synthesized PNOG and PNEHG homopolymers with similar DP_n_ and performed wide-angle X-ray diffraction (WAXD) measurements on the bulk samples that recrystallized from the melt. Figure 6a,b show the WAXD results for the PNOG (DP_n_ = 103) and PNEHG (DP_n_ = 100) homopolymers. The samples were first thermally annealed at *T* = 200 °C (far above their isotropic melting transition temperature)^40^ for 30 min under vacuum, subsequently cooled, and isothermally recrystallized at 45 °C. Note that such temperature is far below the transition temperature from isotropic melt to LC mesophase (i.e., *T*_LC_ ≈ 152 °C) for PNOG and PNEHG.^40,43^ PNOG homopolymer was found to exhibit typical reflection peaks in the WAXD profile due to the side-by-side and face-to-face stackings of the board-like molecules, consistent with those observed for the PNMG-*b*-PNOG microflowers in methanol. By contrast, the PNEHG homopolymer exhibits the primary diffraction peak at q* = 0.50 Å^–1^ and multiple higher order peaks located at √3q*, √4q*, and √7q*, respectively, along with a broad amorphous peak near *q* = 1.2 Å^–1^, which is likely to arise from the interchain distance among the 2-ethyl-1-hexyl side chains. This result agrees well with the GIWAXD analysis for the PNMG-*b*-PNEHG nanosheets formed in solution. The molecular packing of PNEHG bearing long and branched *N*-alkyl substituents differs significantly from that of PNOG with long and linear *N*-alkyl substituent both in the bulk and in solution aggregates. Unlike PNOG chains that preferentially adopt a board-like geometry with all the linear *n*-octyl side chains aligned in the same plane, the greater steric hindrance of the bulky *racemic* 2-ethyl-1-hexyl side chains makes it energetically unfavorable for the PNEHG to adopt a planar geometry. Instead, the PNEHG molecules adopt a rod-like geometry with an extended backbone conformation, which allow the side chains to orient outwardly along the backbone, thereby minimizing steric repulsion among the bulky branched *n*-alkyl substituents.

After clarifying the differences in molecular geometry and packing between PNOG and PNEHG, we now discuss how they affect the final solution morphology of PNMG-*b*-PNOG and PNMG-*b*-PNEHG self-assemblies. As shown in Figure 6c, a single PNOG board-like molecule in the crystalline lattice contains three different facets: the main face of PNOG that comprised both a backbone and *N*-aliphatic side chain, the surface comprised PNOG backbone chain ends, and the surface comprised only *N*-aliphatic side chain ends, which are perpendicular to the crystallographic *a*-, *b*-, and *c*- axes of the PNOG molecule, respectively. Since PNOG blocks are covalently linked with the corona-forming PNMG blocks in the diblock copolypeptoids, it is reasonable that the PNOG crystals tend not to grow along the crystallographic *b-*axis. This is consistent with the time-dependent SAXS/WAXS results (Figure 2), which show that the crystalline growth of the core-forming PNOGs is two-dimensional. We therefore postulate that the core thickness of the PNMG-*b*-PNOG nanoribbons is determined by the crystalline dimension along the *b-*axis, while the other two axes determine the lateral dimension of the nanoribbon core (Figure 7a). As shown in Figure 1b, the total thickness of the PNMG-*b*-PNOG nanoribbons in the dry state is 11 ± 1 nm (which also contains the collapsed PNMG block) and is only ∼39% of the fully extended backbone length of PNOG adopting an all-*cis*-amide conformation, indicating the intramolecular folding of long polypeptoid backbones within the nanoribbons is inevitable.

Based on AFM results (Figure 1b and Figure S7a), the average length of the nanoribbons (*ca.* 500 ± 50 nm) is approximately 4–5 times larger than their width (*ca.* 120 ± 30 nm). Although it remains unclear whether the length of the nanoribbon is due to face-to-face packing (along the *a*-axis) or side-by-side packing (along the *c*-axis) of PNOG, the above results suggest that the tendency for the PNOG core to grow along one axis is 4–5 times higher than the other axis. As demonstrated by this work and other previous studies,^44,45,47,61^ 2D nanostructures assembled from the diblock copolypeptoids bearing board-like crystallizable blocks from solution usually appear nonsymmetrical, such as ribbon-like or rectangular shapes, rather than forming a symmetrical 2D geometry. This is mainly due to the preferential growth along one of the crystallographic axes during the 2D crystallization, which is dictated by the disparate inter- or intramolecular interactions and polymer–solvent interactions along different crystallographic axes.

By contrast, when the PNEHG backbone adopts a helical conformation, which allows the bulky *racemic* 2-ethyl-1-hexyl side chains to be evenly distributed around the backbone (Figure 6c), the rod-like PNEHG blocks would afford identical inter- or intramolecular interactions and polymer–solvent interfacial interactions in the radial direction of the rods. This would favor the columnar hexagonal packing of PNEHG segments, leading to the formation of large hexagonal nanosheets that possess a symmetrical 2D geometry (Figure 7). The symmetrical hexagonal nanosheets formed by PNMG-*b*-PNEHG mesogens are highly unusual and rarely observed by crystallization or CDSA of typical crystalline polymers (e.g., polyethylene and polycaprolactone),^62−64^ which tend to favor anisotropic crystallization, thus yielding asymmetric (or elongated) hexagonal nanosheets. Based on the bulk density of PNEHG (0.97 g/cm^3^),^51^ the theoretical molecular volume of a single PNEHG block (DP_*n*_ = 101) is estimated to be 29.2 nm^3^. Thus, when a rod-like PNEHG block is fitted in a columnar hexagonal lattice with a *d*-spacing of 1.26 nm (which corresponds to the closest distance between two laterally stacked rods obtained by WAXS), the total length of the unfolded PNEHG rod is then estimated to be 21.2 nm. However, according to the model fitting of the SAXS curve (Figure 4c), the averaged core thickness of the PNMG-*b*-PNEHG hexagonal nanosheets is found to be 11.3 ± 0.2 nm, which is only ∼50% of the estimated total length of an unfolded PNEHG rod in a hexagonal columnar phase. This result indicates that the rod of PNEHG must be either (i) self-folded within the core region or (ii) tilted with an angle of ∼60° with respect to the normal direction of the nanosheet basal plane, similar to the packing arrangement of LC molecules in a smectic C phase.^65,66^ According to previous theoretical and experimental studies on rod–coil block copolymers (e.g., helical poly(hexyl isocyanate)-based rod–coil block copolymers), it is expected that the tilting of the core-forming rod-like molecules would increase the interfacial area between core and corona domains, thereby reducing the entropic penalty associated with the stretching of coil-like blocks in the corona.^65−68^ However, the GIWAXD results for the dried PNMG-*b*-PNEHG nanosheets on a Si substrate (Figure 5) show no significant tilting of the rod-like PNEHG, as evidenced by the shape of the primary peak located at *q*_*xy*_ = 0.5 Å^–1^. This may be due to a change of the PNEHGs’ molecular orientation upon solvent removal. The detailed chain conformation and molecular orientation of PNEHG-based self-assemblies will be investigated in our future efforts.

We will also discuss the effect of side chain branching on the self-assembly kinetics and pathways in solution. As evidenced by the time-dependent SAXS/WAXS and AFM results, the self-assembly of PNMG-*b*-PNOG in dilute solution is relatively sluggish, which involves multiple stages including the early micellization of PNMG-*b*-PNOG unimers and the later growth of 2D nanoribbons via crystallization-induced fusion and reorganization of initial amorphous micelles. With an initial polymer concentration of *c* = 5 mg/mL, the entire solution self-assembly process of PNMG-*b*-PNOG takes a few hundreds of minutes to complete (Figure 2). When the initial concentration is lower at *c* = 0.5 mg/mL, the completion of the self-assembly process requires at least half a day or even longer time (Figure 3). Note that the growth of nanoribbons and the formation of microflowers seem to occur simultaneously (Figure 3). By contrast, the self-assembly of PNMG-*b*-PNEHG hexagonal nanosheets in a 5 mg/mL solution via a liquid-crystalline driving force was completed almost immediately upon cooling below the clearing temperature, evidenced by little change of S/WAXS scattering profiles with time (Figure S14). Even in the case of dilute concentration (i.e., *c* = 0.5 mg/mL), the solution turned cloudy within seconds upon cooling below the clearing point, suggesting the occurrence of rapid solution aggregation. We postulate that the differences in molecular geometry and intermolecular packing between PNOG and PNEHG are key factors that dictate the kinetics of their respective self-assembly in solution. For the PNMG-*b*-PNOG microflowers composed of stacked nanoribbons, the formation of a long-range-ordered crystal lattice in the nanoribbons is likely governed by the epitaxial 2D crystalline growth of board-like PNOG segments during a micellar fusion/reorganization process. By contrast, the PNEHG blocks bearing branched *N*-aliphatic side chains adopt a rod-like molecular geometry that favors the intermolecular packing into a columnar hexagonal LC mesophase with short-range ordering. The formation of the mesophase within the micellar core occurs much more rapidly relative to that of the crystalline micellar core presumably due to the less defined molecular packing structure in the former relative to the latter.^5,69^ Further studies on the solution self-assembly of PNMG-*b*-PNOG and PNMG-*b*-PNEHG under different isothermal conditions are currently in progress.

Based on the time-dependent X-ray scattering and AFM results (Figures 2 and 3), it is evident that the formation of microflower nanostructures involves a hierarchical self-assembly of PNMG-*b*-PNOG molecules by a nucleation-and-growth mechanism. The PNMG-*b*-PNOG molecules first associate to form amorphous spherical micelles in methanol owing to the high solvophobic content of the block copolymers (i.e., 73% volume fraction of the PNOG segment). Further aggregation of the amorphous micelles followed by the onset of crystallization (i.e., crystal nucleation) resulted in the formation of flower petal junction. Note that the precise structure of the flower petal junction remains elusive due to the challenge to cleanly isolate this intermediate species for characterization. Following the formation of crystal nuclei, the growth of the flower petals (i.e., nanoribbons subunits) occurs by the addition of the PNMG-*b*-PNOG molecules from the amorphous micelles to the crystallization front following a 2D crystallization kinetics.

**Figure 8:**
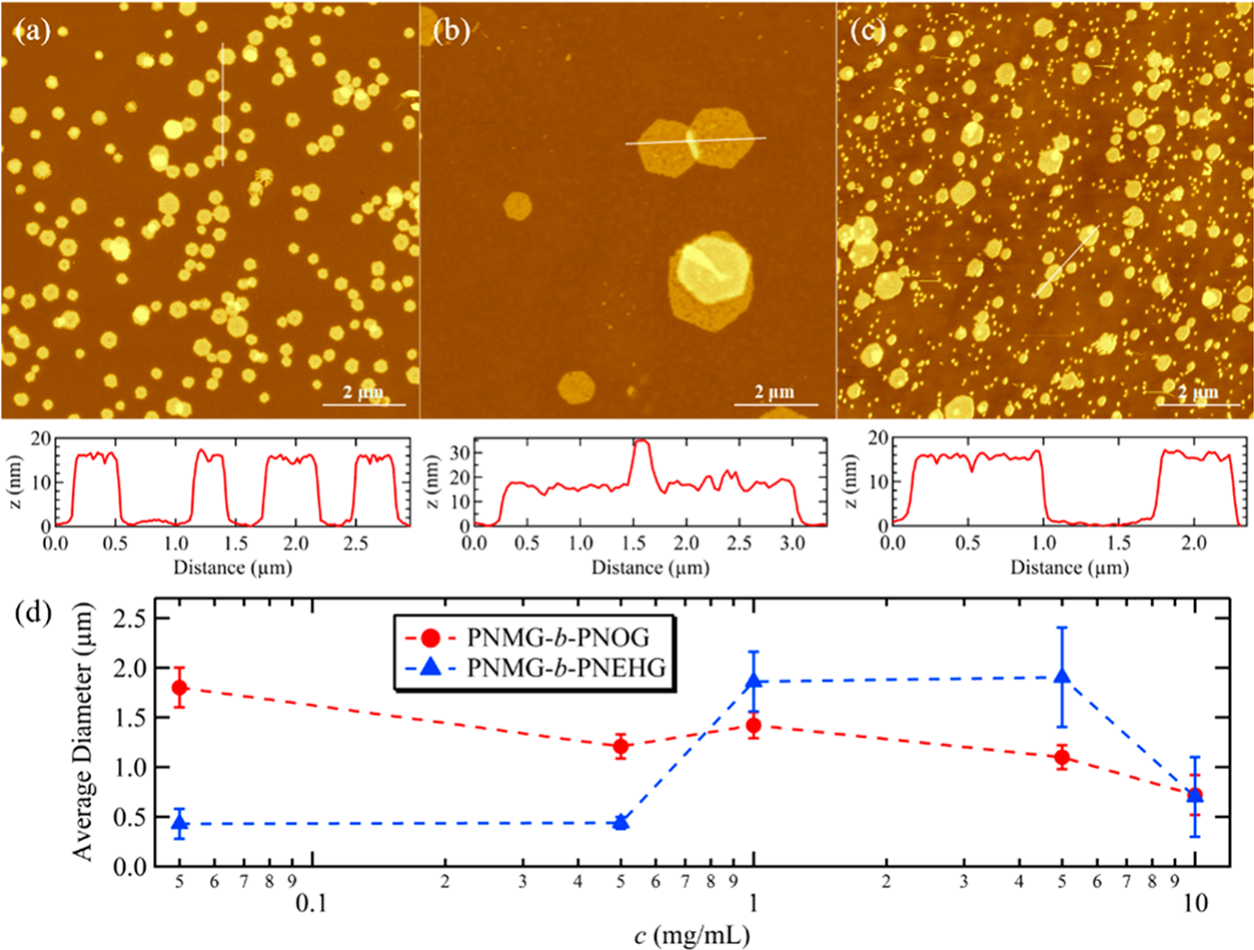
Representative AFM height images for PNMG-*b*-PNEHG hexagonal sheets self-assembled with an initial concentration of (a) 0.5, (b) 1, and (c) 10 mg/mL, respectively. The height profiles along the white lines obtained from AFM cross-sectional analysis are shown below each image. (d) Average sizes of PNMG-*b*-PNOG microflowers and PNMG-*b*-PNEHG nanosheets versus initial solution concentration (*c*).

Finally, we show the effect of initial polymer concentration on the final solution morphology. As aforementioned, the microflower morphology of the PNMG-*b*-PNOG was unaffected by the initial solution concentration within the dilute regime (*c* ≤ 5 mg/mL) (Figure S8). On the basis of AFM and TEM images, we found that the average size of the microflowers decreases with increasing of the initial polymer concentration at *c* ≤ 10 mg/mL (Figure 8d). For *c* = 10 mg/mL, the final PNMG-*b*-PNOG self-assembled structures are no longer flower-like with distinct petals but appear as large fuzzy spheres with an average diameter of a few hundreds of nanometers (Figure S15), which is smaller relative to the microflowers formed at lower concentrations. As the formation of PNMG-*b*-PNOG microflowers occurs by a nucleation-and-growth process, a higher initial polymer concentration can give rise to a significant increase in the number of nucleation sites, thereby resulting in a reduced average size of the final assemblies.

By contrast, the concentration dependence of the final size of the PNMG-*b*-PNEHG nanosheets by LCDSA showed a bell-shaped curve: the average diameter of the nanosheets increases with increasing of the initial solution concentration until reaching a maximum value, then decreases as the concentration was further increased. For *c* = 0.5 and 10 mg/mL, the average dimensions of the resulting PNMG-*b*-PNEHG nanosheets are decreased by nearly 4 times relative to those observed for *c* = 1 and 5 mg/mL. Nevertheless, the thickness and symmetrical hexagonal shape of the nanosheets remain nearly unaffected by the initial polymer concentration (Figure 8). The origin of the bell shape dependence of the nanosheet diameter on the initial polymer concentration remains unclear presently. Efforts focused on understanding the formation mechanisms for the hexagonal nanosheets will be pursued in the future, which will shine a light on the origin of the bell-shaped relationship between the nanosheet diameter and the initial polymer concentration.

## Conclusions

The role of side chain branching on the solution self-assembly of amphiphilic diblock copolypeptoids (i.e., PNMG-*b*-PNOG and PNMG-*b*-PNEHG) has been investigated by a combination of X-ray scattering and microscopic imaging analysis. With linear *n*-octyl side chains, PNOG segments adopt a board-like molecular geometry and can stack face-to-face (along the crystallographic *c*-axis) and side-by-side (along the crystallographic *a*-axis) simultaneously. This enables the two-dimensional and anisotropic crystallization of the core-forming PNOG blocks, resulting in the formation of PNMG-*b*-PNOG nanoribbons that radially stacked within the hierarchical microflowers. By contrast, PNEHGs bearing branched *N*-aliphatic side chains, i.e., *racemic* 2-ethyl-l-hexyl side chains, adopt a rod-like molecular geometry with an extended backbone conformation with the bulky *N*-2-ethyl-1-hexyl side chains radially and outwardly displayed along the backbone. Even though the molecular arrangement of PNEHG lacks long-range ordering, the rod-like PNEHG block can pack into a columnar hexagonal mesophase, which drives the formation of PNMG-*b*-PNEHG symmetric hexagonal nanosheets in methanol.

It was also found that the hierarchical self-assembly of PNMG-*b*-PNOG is relatively sluggish and involves the assembly of multilevel building blocks in a stepwise fashion, including early micellization and fusion/reorganization of the initial micelles into elongated nanoribbons via CDSA. While the hierarchical self-assembly mechanism of these microflowers with radially arranged nanoribbons remains somewhat ambiguous, the formation of elongated nanoribbons (i.e., flower petals) is induced by the 2D crystallization of PNOG due to more favored molecular packing along one of the crystallographic axes. By contrast, the formation of PNMG-*b*-PNEHG hexagonal nanosheets via the LCDSA process is rapid relative to that of the PNMG-*b*-PNOG microflowers, which is attributed to the less defined molecular packing of PNEHG segments within the mesophasic micellar core relative to that of the PNOG segments in the corresponding crystalline micellar core. Furthermore, we demonstrated that the lateral dimensions of PNMG-*b*-PNEHG hexagonal nanosheets can be manipulated from nanosize to microsize by tuning the initial polymer concentration within the dilute regime, while the thickness of hexagonal nanosheets remains unaffected by the concentration. This study highlights the impact of N-substitution architecture on the molecular packing and solution self-assembly of coil-crystalline diblock copolypeptoids, which may serve as a crucial parameter in the rational design of polypeptoid-based nanostructures. The formation of 2D hexagonal nanosheets of diblock copolypeptoids with tunable lateral dimensions induced by LCDSA also shed new light on the creation of highly symmetric 2D nano/microscale materials for a wide range of applications.

## Supporting Information Available

Materials and Methods, schematics of crystalline packing of comb-shaped polypeptoids, ^1^H and ^13^C{^1^H} NMR spectra and SEC-dRI chromatograms of the PNMG-*b*-PNOG and PNMG-*b*-PNEHG block copolymers, TEM/AFM images for the PNMG-*b*-PNOG and PNMG-*b*-PNEHG block copolymers, additional SAXS/WAXS results for the PNMG-*b*-PNOG and PNMG-*b*-PNEHG at different waiting times, high-temperature SAXS/WAXS results for PNMG-*b*-PNOG and PNMG-*b*-PNEHG, SAXS analysis for the PNMG-*b*-PNOG sample at time = 0, core thickness distributions of PNMG-*b*-PNEHG nanosheets, DSC thermograms of PNMG-*b*-PNOG and PNMG-*b*-PNEHG (PDF)
