# Supplementary material for: Modulating the Molecular Geometry and Solution Self-Assembly of Amphiphilic Polypeptoid Block Copolymers by Side Chain Branching Pattern
Source: J Am Chem Soc. 2021 Apr 6;143(15):5890–902. doi: 10.1021/jacs.1c01088 (PMC8154532; doi:10.1021/jacs.1c01088)
Supplement: Supplementary file 1 — ja1c01088_si_001.pdf [file ja1c01088_si_001.pdf]

Supplementary Information

Modulating the Molecular Geometry and Solution Self-Assembly of Amphiphilic Polypeptoid Block Copolymers by Side Chain Branching Pattern

Liying Kang,[1] Albert Chao,[2] Meng Zhang,[2] Tianyi Yu,[2] Jun Wang,[1] Qi Wang,[1] Huihui Yu,[1] Naisheng Jiang[1,2] and Donghui Zhang[2*]*

[1.] School of Materials Science and Engineering, University of Science and Technology Beijing, Beijing 100083, China

[2.] Department of Chemistry and Macromolecular Studies Group, Louisiana State University, Baton Rouge, Louisiana 70803, United States

[*] *Corresponding to*: dhzhang@lsu.edu and naishengjiang@ustb.edu.cn

General Considerations. Deuterated dichloromethane (CD_2Cl_2, 99.8 atom % D) was purchased from Cambridge Isotope Laboratories. All other chemicals were purchased from Sigma Aldrich and used as received unless otherwise noted. All solvents used in polymerization were purified by passing through alumina columns under argon. ^{1}H NMR spectra and ^{13}C{^{1}H} NMR spectra were obtained using a Bruker AV-400 Nanobay spectrometer (400 MHz for ^{1}H NMR)) and a JEOL JNM_ECA400 spectrometer (400 MHz for ^{13}C NMR) at 25 °C. Chemical shifts (δ) given in parts per million (ppm) were referenced to protio impurities of deuterated dichloromethane (CD_2Cl_2). *N*-methyl, *N*-octyl and *N*-2-ethyl-1-hexyl glycine derived *N*-carboxyanhydride (Me-NCA, Oct-NCA and EtHex-NCA) monomers were synthesized according to the established procedures.[S1-S3]

Synthesis of PNMG-*b*-PNOG Diblock Copolypeptoids. In the glovebox, Me-NCA ($[M_1]$) (80 mg, 0.7 mmol, $[M_1]_0 = 1$ M) was dissolved in anhydrous DCM (0.69 mL) in a vial. A known volume of benzylamine (I) stock solution in anhydrous DCM (14 μL, 7 μmol, $[I]_0 = 0.5$ M, $[M_1]_0:[I]_0 = 100:1$) was added to the monomer solution using a micro-syringe. The reaction proceeded at room temperature under nitrogen for 24 h to reach a full conversion prior to the addition of the second monomer (Oct-NCA). A stock solution of Oct-NCA ($[M_2]$) (0.5 mL, 0.7 mmol, $[M_2]_0 = 1.4$ M, $[M_2]_0:[I]_0 = 100:1$) was added to the above reaction mixture and was heated at 50 °C for an additional 48 h to reach a complete conversion. The volatiles were removed under vacuum to afford a crude polymer which was purified by re-dissolution in DCM followed by addition of excess hexanes twice to precipitate out the polymer. The polymer was isolated by centrifugation, filtration and dried under vacuum to yield a white powder. ^{1}H NMR data (CD_2Cl_2, 400 MHZ) δ (ppm): 0.91 ($C\boldsymbol{H}_3(CH_2)_6CH_2$-); 1.32 ($CH_3(C\boldsymbol{H}_2)_5CH_2CH_2$-); 1.53 ($CH_3(CH_2)_5C\boldsymbol{H}_2CH_2$-); 2.93-3.05 ($C\boldsymbol{H}_3N$-); 3.38 ($CH_3(CH_2)_5CH_2C\boldsymbol{H}_2$-); 4.05-4.27 ($NCOC\boldsymbol{H}_2$-); 7.34 ($C_6\boldsymbol{H}_5$-). ^{13}C{^{1}H} NMR data (CD_2Cl_2, 100 MHZ) δ (ppm): 13.9 ($\boldsymbol{C}H_3(CH_2)_6CH_2$-); 22.7 ($CH_3\boldsymbol{C}H_2(CH_2)_5CH_2$-); 27.1 ($CH_3CH_2\boldsymbol{C}H_2(CH_2)_4CH_2$-); 29.4 ($CH_3(CH_2)_2\boldsymbol{C}H_2(CH_2)_3CH_2$-); 29.5 ($CH_3(CH_2)_3\boldsymbol{C}H_2(CH_2)_2CH_2$-); 31.9 ($CH_3(CH_2)_5\boldsymbol{C}H_2CH_2$-); 35.6 ($\boldsymbol{C}H_3N$-); 48.5 ($CH_3(CH_2)_6\boldsymbol{C}H_2$-); 49.4-50.4 ($NCO\boldsymbol{C}H_2$-); 169.2 ($N\boldsymbol{C}OCH_2$-). The ^{1}H NMR spectra and ^{13}C{^{1}H} NMR spectra of PNMG-*b*-PNOG are shown in Figure S2 and S3, respectively. The block copolymer composition was determined by the end-group analysis using the corresponding ^{1}H NMR spectrum. Specifically, the number-averaged degree of polymerization for the PNOG segment was determined by integrating the methyl protons of PNOG segment at 0.80 ppm (a in Figure S2) relative to the phenyl protons of the

benzylamide end-group centered at ~7.2 ppm (l-n in Figure S2). The number-averaged degree of polymerization of the PNMG segment was determined from the integration of methylene protons for PNOG at 3.22 ppm (h in Figure S2) and methyl protons for PNMG at 2.93-2.77 ppm (k in Figure S2) subtracted by the integration of methylene protons on the backbones for both PNMG and PNOG segments at 4.15-3.80 ppm (i, j in Figure S2).

Synthesis of PNMG-*b*-PNEHG Diblock Copolypeptoids. In the glovebox, EtHex-NCA ($[M_2]$) (100 mg, 0.47 mmol, $[M_2]_0 = 0.2$ M) was dissolved in anhydrous THF (0.50 mL) in a vial. A known volume of benzylamine (I) stock solution in anhydrous acetonitrile (19 µL, 4.7 µmol, $[I]_0 = 0.5$ M, $[M_2]_0:[I]_0 = 10:1$) was added to the monomer solution using a micro-syringe. The vial was sealed, and the reaction mixture was stirred at 70 °C for 24 h to reach a complete conversion. THF was removed from the reaction mixture under vacuum. The resulting PNEHG polymer solid were re-dissolved in DCM (0.40 mL), to which a stock solution of Me-NCA (0.5 mL, 0.47 mmol, $[M_1]_0 = 0.94$ M, $[M_1]_0:[I]_0 = 100:1$) in DCM was added in entirety. The reaction mixture was stirred at room temperature for additional 24 h under nitrogen. The volatiles were then removed under vacuum to afford a crude polymer, which was purified by re-dissolution in DCM followed by addition of excess hexanes twice to precipitate out the polymer. The polymer was isolated by centrifugation, filtration and dried under vacuum. (Note: EtHex-NCA is highly soluble in THF but not in DCM, whereas Me-NCA is very soluble in DCM but not THF. The PNMG segment is soluble in DCM but not in THF, whereas the PNEHG segment is soluble in both DCM and THF. As a result, to ensure a homogeneous solution polymerization, we synthesized the PNMG-*b*-PNEHG block copolymer by sequential polymerization of EtHex-NCA and Me-NCA involving a change of solvent). ^{1}H NMR data (CD_2Cl_2, 400 MHZ) δ (ppm): 0.91-0.92 ($CH_3(CH_2)_3CH((CH_2)(C\boldsymbol{H}_3))CH_2$- & $C\boldsymbol{H}_3(CH_2)_3CH(C_2H_5)CH_2$-); 1.30 ($CH_3(CH_2)_3CH((C\boldsymbol{H}_2)(CH_3))CH_2$- & $CH_3(C\boldsymbol{H}_2)_3CH(C_2H_5)CH_2$-); 1.62 ($CH_3(CH_2)_3C\boldsymbol{H}(C_2H_5)CH_2$-); 2.9-3.1 ($CH_3(CH_2)_3CH(C_2H_5)C\boldsymbol{H}_2$- & $C\boldsymbol{H}_3N$-); 4.0-4.3 (-$NCOC\boldsymbol{H}_2$-); 7.34 ($C_6\boldsymbol{H}_5$-). $^{13}C\{^1H\}$ NMR data (CD_2Cl_2, 100 MHZ) δ (ppm): 10.5 ($CH_3(CH_2)_3CH((CH_2)(\boldsymbol{C}H_3))CH_2$-); 13.9 ($\boldsymbol{C}H_3(CH_2)_3CH(C_2H_5)CH_2$-); 23.2 ($CH_3\boldsymbol{C}H_2(CH_2)_2CH(C_2H_5)CH_2$-); 23.9 ($CH_3CH_2\boldsymbol{C}H_2CH_2CH(C_2H_5)CH_2$-); 28.8 ($CH_3(CH_2)_3CH(\boldsymbol{C}H_2(CH_3))CH_2$-); 30.7 ($CH_3(CH_2)_2\boldsymbol{C}H_2CH(C_2H_5)CH_2$-); 36.7 ($CH_3(CH_2)_3\boldsymbol{C}H(C_2H_5)CH_2$-); 37.5($\boldsymbol{C}H_3N$-); 49.2 ($CH_3(CH_2)_3CH(C_2H_5)\boldsymbol{C}H_2$-); 50.4-51.7 ($NCO\boldsymbol{C}H_2$-); 169.8 (-$N\boldsymbol{C}OCH_2$-). The ^{1}H NMR spectra and

$^{13}C\{^1H\}$ NMR spectra of PNMG-*b*-PNEHG are shown in Figure S4 and S5, respectively. The block copolymer composition was determined by the end-group analysis using the corresponding ^1H NMR spectrum. Specifically, the number-averaged degree of polymerization for the PNEHG segment was determined by integrating the methyl protons of PNEHG segment at 0.80 ppm (a, g in Figure S4) relative to the phenyl protons of the benzylamide end-group centered at ~7.2 ppm (l-n in Figure S4). The number-averaged degree of polymerization of the PNMG segment was determined from the integration of methylene protons for PNEHG at 3.22 ppm (h in Figure S4) and methyl protons for PNMG at 2.93-2.77 ppm (k in Figure S4) subtracted by the integration of methylene protons on the backbones for both PNMG and PNEHG segments at 4.15-3.80 ppm (i, j in Figure S4).

Size-Exclusion Chromatography (SEC). SEC analysis of the PNMG-*b*-PNEHG and PNMG-*b*-PNOG samples were performed using a Tosoh EcoSEC Model HLC-8240 GPC system (Tosoh Bioscience LLC, Japan) equipped with a Tosoh Dual-Flow refractive index (RI) detector and autosampler. Two sample columns were installed with TSKgel SuperAWM-H 6mm I.D. × 15 cm, 9 μm packing beads and the reference column was installed with one TSKgel SuperH-RC 6mm I.D. × 15 cm, 4 μm packing beads. HFIP with 0.05 M potassium trifluoroacetate (KTFA) was used as the eluent at a flow rate of 0.35 mL/min. The temperature of column and detector was set at 35 °C. Poly(methyl methacrylate) (PMMA, Mp = 0.613, 1.35, 2.04, 6.39, 24.3, 67.7, 107, 183, 421, 939 kDa) were used as the calibration standards.

Preparation of PNMG-*b*-PNEHG and PNMG-*b*-PNOG Self-assemblies in Methanol Solutions. Diblock copolypeptoids polymer solutions were prepared by dissolving the polymers in methanol with the concentration of 5 mg/mL at 70 °C (PNMG-*b*-PNOG) and 100 °C (PNMG-*b*-PNEHG) for 10 min until clear followed by cooling down to room temperature within 10 min. The solutions were then stored at room temperature for 24 h to complete the self-assembly process prior to any further characterization. For cryo-TEM and AFM measurements, the final self-assembled 5 mg/mL solutions were further diluted to either 0.5 or 1.0 mg/mL using methanol to ensure good imaging quality. PNMG-b-PNEHG and PNMG-b-PNOG solutions with different initial concentrations were prepared by following the same annealing procedure, unless otherwise stated.

Cryo- and Regular Transmission Electron Microscopy (Cryo-TEM and TEM). Cryo-TEM imaging was done on a FEI G2 F30 Tecnai TEM operated at 200 kV. PNMG-*b*-PNOG or PNMG-*b*-

PNEHG solutions (0.5 or 1 mg/mL in concentration) were prepared by direct dilution of the 5 mg/mL self-assembled solutions, respectively. 5 µL of the diluted polymer solution was applied to a 200-mesh lacey carbon grid (Electron Microscopy Sciences) mounted on the FEI Vitrobot. The excess liquid was removed by blotting the grid by filter papers for 2 seconds to form a thin sample film. The grid was then quickly plunged into liquid ethane chilled by liquid nitrogen to vitrify the sample film. The vitrified sample was finally transferred onto a single tilt cryo specimen holder for imaging. Regular TEM was conducted on a JEOL-2200FS TEM operated at 200 kV. The sample for the regular TEM analysis was prepared by adding 10 µL of the diluted polymer solution onto a 200-mesh ultra-thin micro grid followed by blotting with a filter paper and drying at room temperature. The TEM images were generated using TIA software (Thermo Fisher Scientific) and image analysis was conducted using the ImageJ software (http://imagej.net/).

Atomic Force Microscopy (AFM). Atomic force microscopy (AFM) (Bruker Dimension Icon, Hamburg, Germany) was used to study the morphologies of self-assembled polypeptoid nanostructures. The PNMG-*b*-PNOG or PNMG-*b*-PNEHG methanol solutions after self-assembly were spin coated onto piranha solution-cleaned Si (100) substrates. A peak-force tapping mode (*i.e.*, ScanAsyst mode) was conducted under ambient conditions using a ScanAsyst-air probe with a frequency of ~ 70 kHz and a spring constant of 0.4 N/m. The images were captured with a scan rate of ~ 1 Hz and a scanning density of 512 lines per frame. The AFM image analysis was conducted using the Gwyddion software (version 2.47, http://gwyddion.net/).[S4]

Differential Scanning Calorimetry (DSC). DSC analyses of PNMG-*b*-PNOG and PNMG-*b*-PNEHG samples in solid state were conducted on a heat-flux DSC device (Q2000, TA Instruments, New Castle, DE). For PNOG (DP_n = 103) and PNEHG (DP_n = 100) homopolymers, DSC analyses were conducted on a power-compensated DSC device (PerkinElmer DSC-8000). Solid samples (7 – 12 mg in mass) loaded into aluminum pans were first heated from room temperature to 200 °C at 10 °C/min, then cooled to 0°C at 5 °C/min and reheated to 200 °C at 10 °C/min.

Small/Wide Angle X-ray Scattering (SAXS/WAXS). Synchrotron X-ray measurements were performed at the DND-CAT 5-ID-D beamline at the Advanced Photon Source (Argonne National Laboratory, Argonne, IL) with an X-ray wavelength of 1.37 Å (which is equivalent to the X-ray energy of 9.0 keV). A small-angle and two wide-angle X-ray scattering (SAXS/WAXS) 2D patterns were collected simultaneously on three Rayonix area CCD detectors at sample-to-detector distances of

approximately 8.5, 1.0 and 0.2 m, respectively. The combination of three detectors covers an effective q-range of ~ 0.0015 to 1.7 Å^{-1}, which was calibrated using silver behenate. Samples were measured in a capillary flow cell at room temperature with a 1.5 mm nominal diameter quartz capillary (Charles Supper Company). The inner diameter of the capillary cell is estimated to be 1.47 mm from X-ray scans. The 2D scattering patterns were azimuthally isotropic and were azimuthally integrated around the beam center to produce one-dimensional I(q) vs. q scattering profiles. The final X-ray curves of each sample were obtained by averaging 10 frames from different spots with an exposure time of 1 s per each frame. Note that no radiation damage to the sample was observed with the 1 s exposure time. Absolute intensity calibration was performed by measuring the empty capillary cell as well as the cell filled with Millipore water. To obtain the scattering contribution of solely the polymer nanostructures within the dilute solutions, the scattering contribution of pure methanol was subtracted from the PNMG-*b*-PNEHG solution scattering profiles. The calibration, data reduction and background subtraction were performed by using the Irena SAS macros for Igor Pro.[S5]

Additional *in-situ* high temperature SAXS/WAXS measurements were performed at the Life Science X-ray Scattering (LiX/16-ID) beamline of the National Synchrotron Light Source II (NSLS-II, Brookhaven National Laboratory) with an X-ray wavelength of 0.827 Å (which is equivalent to the X-ray energy of 15.0 keV). A small-angle and two wide-angle X-ray scattering (SAXS/WAXS) 2D patterns were collected simultaneously on three Pilatus detectors at sample-to-detector distances of approximately 3.58, 0.71 and 0.34 m, respectively. The combination of three detectors covers an effective q-range of ~ 0.006 to 3.0 Å^{-1}, which was calibrated using silver behenate. Samples were first loaded into quartz capillary tubes (1.5 mm in nominal diameter, Charles Supper Company). Then the capillary tubes were sealed and positioned onto a temperature-controlled multi-sample holder. For PNMG-*b*-PNOG, the sample contained inside of the sealed capillary tubes was first heated and held at 80 °C for 1 min, then cooled and held at 70 °C for 1 min before taking measurements. For PNMG-*b*-PNEHG, the sample contained inside of the sealed capillary tubes was heated and held at 100 °C for 1 min before taking measurements. Such *in situ* annealing process enables us to probe the aggregation state of polymers in solution at high temperatures, while at the same time, allow us to minimize the change of solution volume due to possible solvent evaporation within the sealed capillary tubes during high temperature measurements. Pure methanol was measured in the same capillary tubes individually

for solvent subtraction. Absolute intensity calibration was performed by measuring capillary tube filled with Millipore water.

Grazing Incidence Wide-Angle X-Ray Diffraction (GIWAXD). Grazing incidence wide-angle x-ray diffraction (GIWAXD) measurements for the self-assembled PNMG-*b*-PNEHG hexagonal nanosheets deposited on Si substrate were carried out at the Complex Materials Scattering (CMS/11-BM) beamline at the National Synchrotron Light Source II (NSLS-II), Brookhaven National Laboratory. Two-dimensional (2D) GIWAXD patterns were collected using a Dectris Pilatus 800k area detector (pixel size = 172 μm × 172 μm). The detector distance was calibrated using a silver behenate standard sample. The incident angle of X-rays was set to 0.10° at a wavelength of λ = 0.918 Å (which is equivalent to the X-ray energy of 13.5 keV) and each sample exposure time was fixed to 120 seconds. Multiple 2D images per sample were collected with offset detector positions to avoid loss of information from the detector gaps. GIWAXD 2D patterns were transformed into 1D profiles along the q_{xy}-direction (horizontal axis) and q_z-direction (vertical axis) using the Nika package for Igor Pro,[S6] with q_{xy} and q_z being the scattering vectors in the parallel and perpendicular directions to the substrate surface, respectively.

The SAXS analysis for PNMG-*b*-PNEHG Hexagonal Nanosheets. The scattering form factor for core-corona disk-shaped micelles (*a.k.a.* block copolymer micelles with round disk-shaped cores) developed by Pedersen and co-workers[S7-S8] was used to fit the SAXS profile of the self-assembled PNMG-*b*-PNEHG nanosheets in solution. The scattering form factor of a single micelle with a compact round disk-shaped core surrounded by Gaussian corona chains at the core surface consists of four different terms: the self-correlation of the disk-shaped core, the self-correlation of the chains, the cross term between the disk-shaped core and chains, and the cross term between different chains, which are expressed in terms of the disk-shaped core thickness (H_c) and lateral diameter (D), as well as the radius of gyration ($R_{g,\text{ chain}}$) of the Gaussian corona chains that are centered at a distance $d_{int}R_{g,\text{ chain}}$ away from the surface of the disk-shaped core (d_{int} is close to unity to mimic non-penetration of the corona chains into the core region) (*vide infra*). Dispersity in H_c was also included in the modeling by a lognormal distribution of core thicknesses with a polydispersity value (σ_{Hc}). Since all samples are at dilute concentration, the interparticle interactions arising from the structure factor can be neglected. Note that even though the PNMG-*b*-PNEHG nanosheets are hexagon-shaped, a good fitting of the SAXS

data using the disk-shaped micellar model suggests that the hexagonal core face can be well approximated as a circular shape.

In the fitting of SAXS data, four independent parameters could be adjusted: the core thickness (H_c), the disk diameter (D), radius of gyration of the corona chains ($R_{g,chain}$), and the polydispersity of core thicknesses (σ_{Hc}). The d_{int} value is fixed to 0.9 based on the previous study by Pedersen and Gerstenberg on block copolymer micelles.[S7] The *SLD* values and molecular volume of PNEHG (V_{core}) and PNMG (V_{corona}) were estimated based on the bulk densities of PNEHG and PNMG homopolymers.[S9-S10] An q-independent background term accountable for the incoherent background scattering from the sample was also included. As we found, the most sensitive fitting parameters that determine the overall shape of the fitting curve are the core thickness (H_c), radius of gyration of the corona chains ($R_{g,chain}$). Note that due to the limited q range in the scattering experiments and size polydispersity of the nanosheets, the value of D cannot be determined unambiguous from the present model fitting thus are not discussed here. The fitting of the SAXS data was performed using the SASfit software, which is available free of charge.[S11]

Theoretical Scattering Model for Disk-shaped Micelles. According to the early description by Pedersen and coworkers,[S8] the scattering form factor of a single micelle with a homogeneous core surrounded by Gaussian corona chains contains four different terms: the self-correlation term of the core, the self-correlation term of the corona chains, the cross term between the core and corona chains, and the cross term between different chains in the corona. The equation can be written as:

$$P_{mic}(q) = N_{agg}^2 \beta_{core}^2 P_{core}(q) + N_{agg} \beta_{corona}^2 P_{corona}(q) + 2N_{agg}^2 \beta_{core} \beta_{corona} S_{core\text{-}corona}(q) + N_{agg}(N_{agg} - 1) \beta_{corona}^2 S_{corona\text{-}corona}(q) \quad \text{(eq. 1)}$$

In this equation, q is the scattering vector, N_{agg} is the aggregation number of the micelle, β_{core} and β_{corona} are the total excess scattering length of the core block and the corona block, respectively. Assuming the core is completely dry, β_{core} and β_{corona} are defined as: $\beta_{core} = (SLD_{core} - SLD_{solvent})V_{core}$ and $\beta_{corona} = (SLD_{corona} - SLD_{solvent})V_{corona}$, where SLD_{core}, SLD_{corona} and $SLD_{solvent}$ are the scattering length densities of the core block (PNEHG), corona block (PNMG) and solvent (methanol), respectively; V_{core} and V_{corona} are the molecular volumes of a single PNEHG chain in the core and a single PNMG chain in the corona, respectively. For a disk core with a diameter D and a thickness H_c, N_{agg} is given by $N_{agg} = \pi D^2 H_c / 4V_{core}$. Based on the reported bulk densities of PNMG and PNEHG, the SLD_{core} and SLD_{corona} values under X-ray were obtained via the equation $SLD = n_e N_A \rho_m r_e / M_w$, where n_e is the

number of electrons per molecule, N_A is the Avogadro's number, ρ_m is the mass density of a material, $r_e = 2.82 \times 10^{-15}$ m is classical electron radius, M_w is the molar mass of the material.

In the limit of $D >> H_c$, the self-correlation term of the core $P_{core}(q)$ for a disk-shaped micellar core can be written as:[S8]

$$P_{core}(q)=\frac{2}{q^2D^2}\left[1-\frac{J_1(2qD)}{qD}\right]\left[\frac{\sin(qH_c/2)}{qH_c/2}\right]^2 \quad \text{(eq. 2)}$$

Where q is the length of scattering vector and D is the disk diameter and H_c is the core thickness, and $J_1(x)$ is the first order Bessel function of the first kind.

In the second term, for the Gaussian chains with a radius of gyration R_g in the corona, $P_{corona}(q)$ is therefore given by the Debye function:[S12]

$$P_{corona}(q) = \frac{2[\exp(-q^2R_g^{\,2}) + q^2R_g^{\,2} - 1]}{q^4R_g^{\,4}} \quad \text{(eq. 3)}$$

According to the description, the Gaussian chains are uniformly distributed at a distance $d_{int}R_g$ away from the surface of the disk core, where d_{int} is close to unity as to mimic non-penetration of the corona chains into the core region. The cross term between the core and corona chains in the third term is approximated by:[S7]

$$S_{core\text{-}corona}(q) = \frac{1 - \exp(-q^2R_g^{\,2})}{q^2R_g^{\,2}}\frac{\sin(qH_c/2)}{qH_c/2}\cos\left[q\left(H_c/2+d_{int}R_g\right)\right]\frac{2}{q^2D^2}\left[1-\frac{J_1(2qD)}{qD}\right] \quad \text{(eq. 4)}$$

In the fourth term, the interference term between the corona chains is given by:[S7]

$$S_{corona\text{-}corona}(q) = \frac{[1 - \exp(-q^2R_g^{\,2})]^2}{q^4R_g^{\,4}}\cos\left[q\left(H_c/2 + d_{int}R_g\right)\right]^2\frac{2}{q^2D^2}\left[1-\frac{J_l(2qD)}{qD}\right] \quad \text{(eq. 5)}$$

In the modelling, the polydispersity of the core thickness has also been considered, in which a LogNormal distribution is assumed:

$$P(H_c) = \frac{1}{\sqrt{2\pi}H_c\sigma_{H_c}}\exp\left[\frac{\ln H_c - \ln\langle H_c\rangle}{-2\sigma_{H_c}^{\,2}}\right] \quad \text{(eq. 6)}$$

where $\langle H_c\rangle$ is average core thickness and σ_{Hc} is the standard deviation of the core thickness.

For dilute concentration, the interparticle interactions arising from the structure factor can be neglected during the model fitting. The final expression for the coherent scattering intensity can then be written as:

$$I(q) = nP_{mic}(q) \qquad \text{(eq. 7)}$$

where n is the number density of particles in the system, which can be described as $n = f_p/N_{agg}(V_{core} + V_{corona})$, where f_p is the volume fraction of diblock copolypeptoids in the solution.

Beaucage Unified Function. We applied a three-level Beaucage unified function[S13] to fit the SAXS profiles of the 5 mg/mL PNMG-*b*-PNOG methanol solutions at 0 min after been cooled down to room temperature, assuming that the solution at 0 min consists of aggregates of early formed spherical micelles that comprised of uncrystallized polymer chains. The Beaucage equation can be written as:

$$I(q) = \sum_{i=1}^{3}\left[G_i \exp\left(-\frac{q^2R_{gi}^2}{3}\right) + B_i\exp\left(-\frac{q^2R_{g(i+1)}^2}{3}\right)\left(\frac{1}{q^*}\right)^{P_i}\right] \qquad \text{(eq. 8)}$$

where

$$q_i^* = q\left[\mathrm{erf}\left(\frac{qR_{gi}}{\sqrt{6}}\right)\right]^{-3}$$

In this case, level 1, 2 and 3 refer to large aggregates of spherical particles, individual spherical particles (*i.e.*, early formed spherical micelles) and uncrystallized polymer chains, respectively.

For individual polymer chains, a simple relationship between the power-law prefactor, B, and the radius of gyration of the coil can be obtained from the Debye equation for polymer coils,[S12] where $B = 2G/R_g^2$. Similarly, a relationship of $B = 81G/(50R_g^4)$ can be obtained from the spherical-shaped particles for the second level.[S14] Here, R_g of the uncrystallized polymer coil (level 3) was fixed to 3.2 nm based on the Guinier plot analysis for the *in situ* high temperature SAXS results at 70 °C (Figure S9a). As shown in Figure S11, the best-fit to the data gives a radius of gyration of 11.5 nm for the early formed spherical micelles (*i.e.*, level 2), corresponding to a radius of 14.8 nm (via $R_g^2 = 3/5\ R^2$) for the spherical-shaped particles. This result is in good agreement with the AFM results for PNMG-*b*-PNOG assemblies at 0 min, which show nanoparticles with an average radius of ~ 15 nm throughout the sample (Figure 3a).

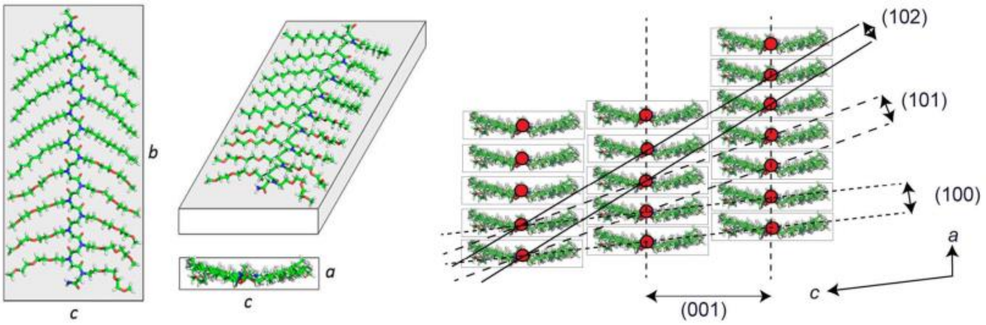

Figure S1. The unit cell dimension and supramolecular assembly of peptoid molecules with all cis-amide backbone conformation in the crystalline state. Note that in the crystalline state, peptoid molecules with N backbone repeating units and S number of carbons on the *n*-alkyl sidechains adopt an energetically favorable all-*cis* backbone conformation, and the unit cell dimensions, namely a, b, and c, follow a universal relationship, in which a = 0.455 nm, b = (0.298N + 0.035) nm and c = (0.186S + 0.55) nm. (Figure reproduced from Greer, D. R.; Stolberg, M. A.; Kundu, J.; Spencer, R. K.; Pascal, T.; Prendergast, D.; Balsara, N. P.; Zuckermann, R. N. Universal Relationship between Molecular Structure and Crystal Structure in Peptoid Polymers and Prevalence of the cis Backbone Conformation. *J. Am. Chem. Soc.* **2018**, *140*, 827-833.).

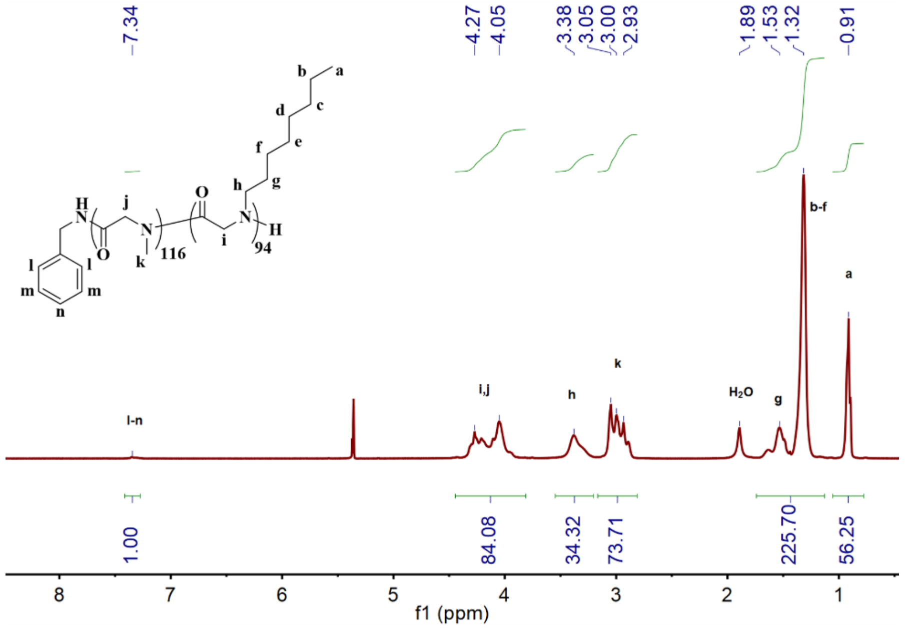

Figure S2. ^{1}H NMR spectrum of PNMG-*b*-PNOG in CD_2Cl_2.

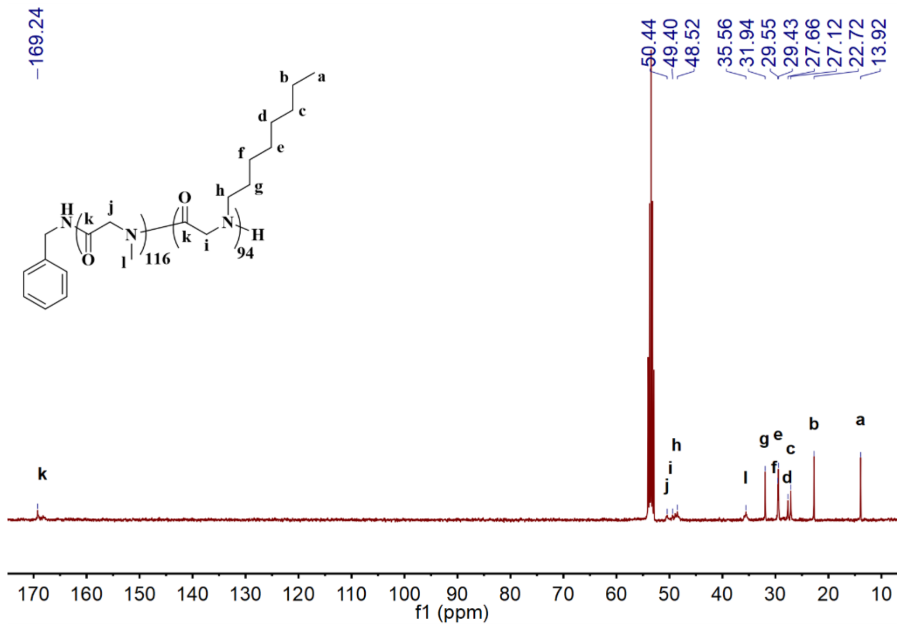

Figure S3. $^{13}C\{^{1}H\}$ NMR spectrum of PNMG-*b*-PNOG in CD_2Cl_2.

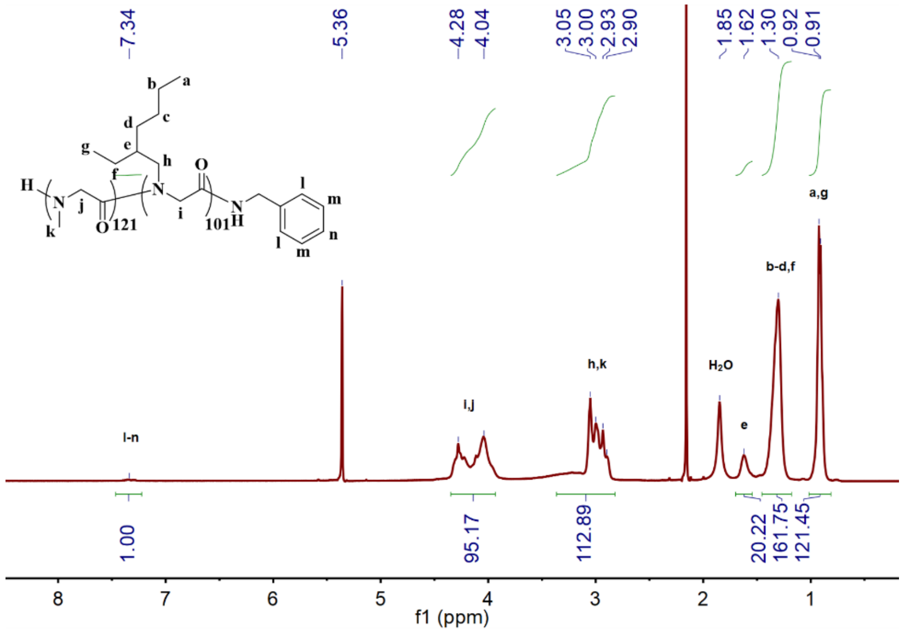

Figure S4. ^{1}H NMR spectrum of PNMG-*b*-PNEHG in CD_2Cl_2.

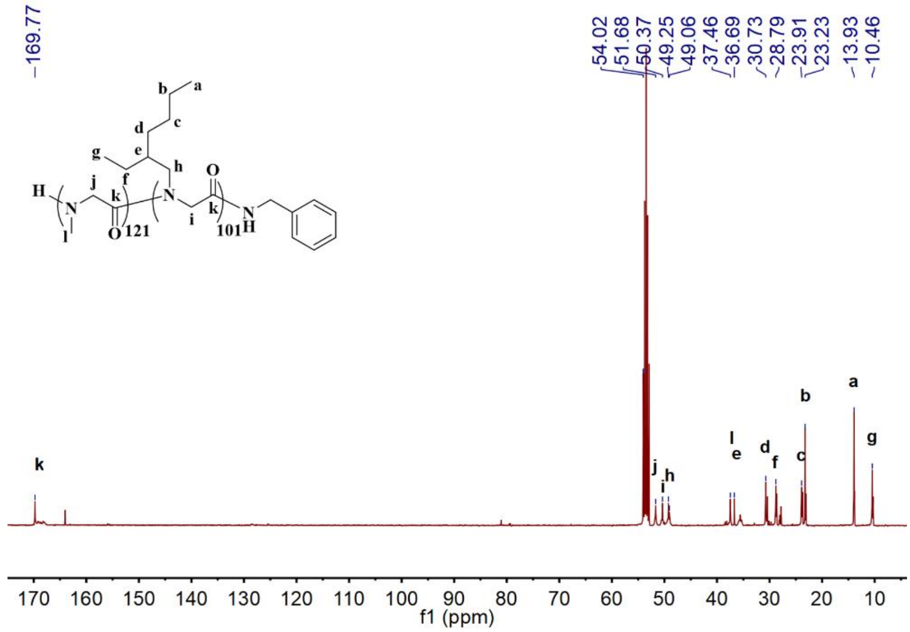

Figure S5. $^{13}C\{^{1}H\}$ NMR spectrum of PNMG-*b*-PNEHG in CD_2Cl_2.

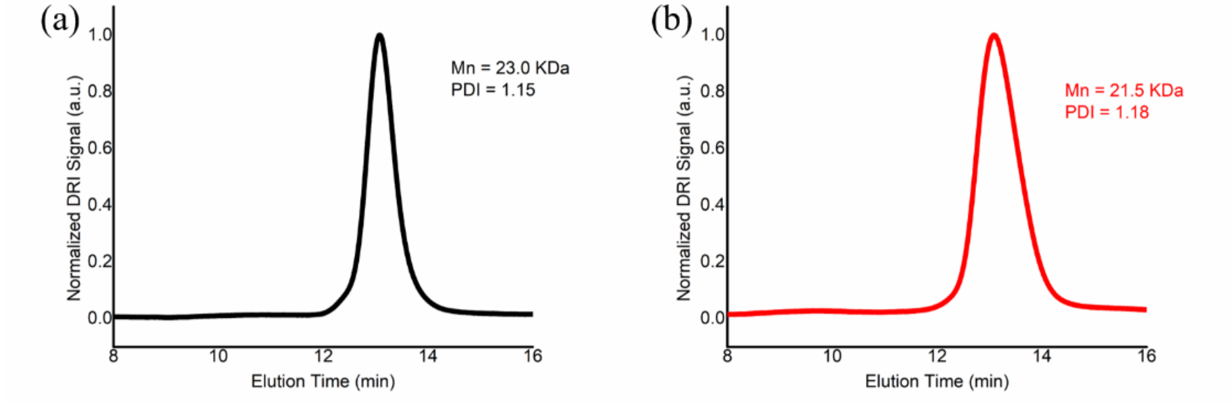

Figure S6. SEC-DRI chromatograms of the (a) PNMG-*b*-PNOG and (b) PNMG-*b*-PNEHG in HFIP with 0.05 M potassium trifluoroacetate (KTFA) at 35 °C. M_n and polydispersity index (PDI) of the polymers were obtained by using a calibration curve constructed from PMMA standards.

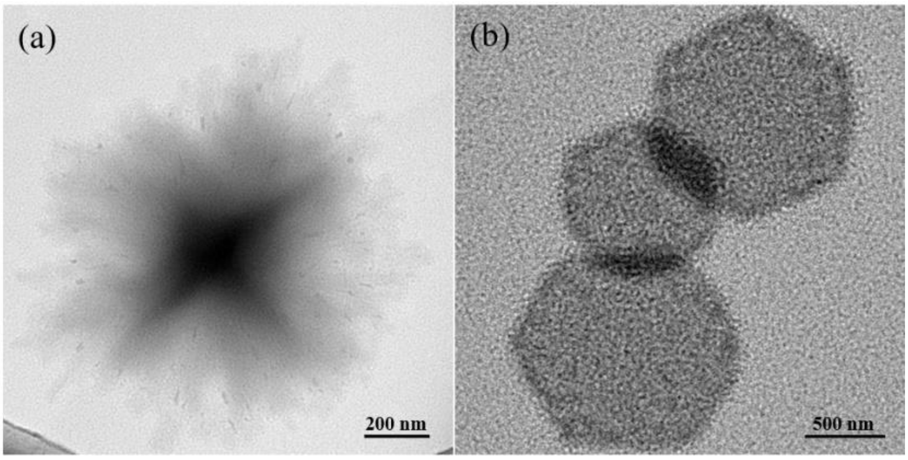

Figure S7. Regular TEM images for (a) PNMG-*b*-PNOG and (b) PNMG-*b*-PNEHG self-assemblies. The samples were prepared by dilution from the original 5 mg/mL solution, and then deposited onto TEM grids. The overall morphologies for PNMG-*b*-PNOG and PNMG-*b*-PNEHG are similar to those captured by cryo-TEM, indicating that regular TEM is experimentally valid in revealing the self-assembled morphology in solution.

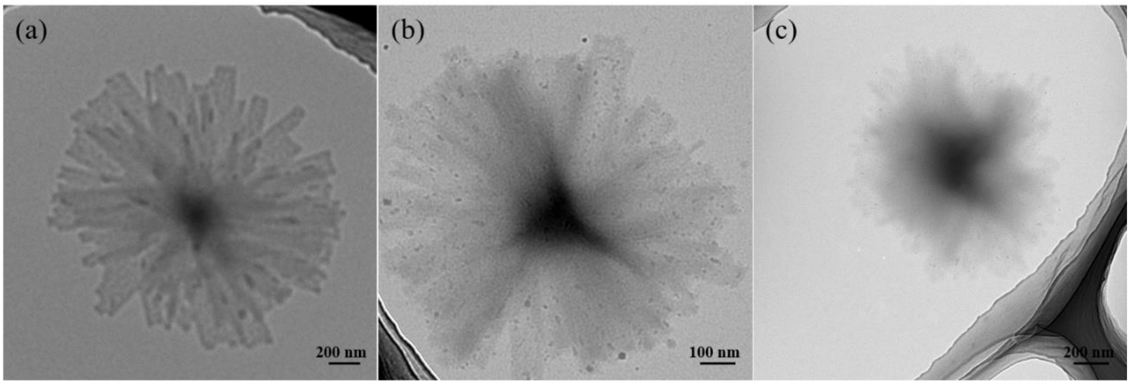

Figure S8. Regular TEM images for PNMG-*b*-PNOG self-assembled from methanol solutions with different initial concentrations: (a) 0.05 mg/mL, (b) 0.5 mg/mL and (c) 5 mg/mL.

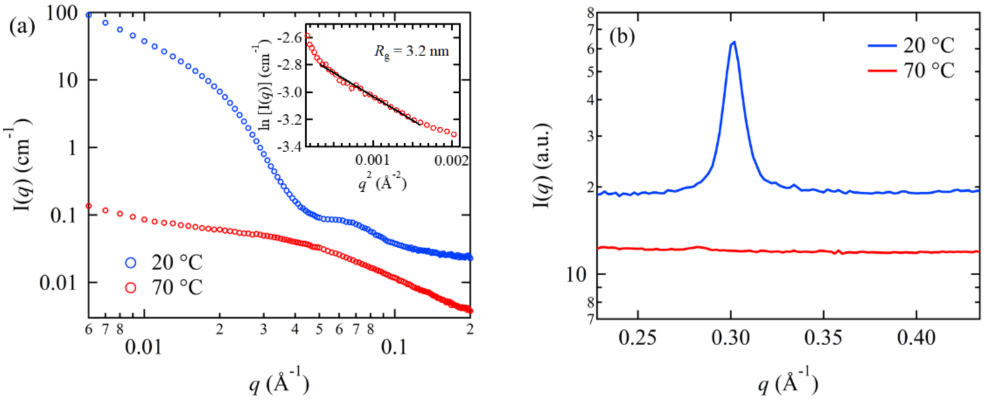

Figure S9. (a) SAXS intensity profiles for the 5 mg/mL PNMG-*b*-PNOG in methanol measured at 70 °C (red circles) and 20 °C (before heating, blue circles) using a sealed capillary tube. For the measurement took at 70 °C, the sample was first heated and held at 80 °C for 1 min, then cooled and held at 70 °C for 1 min before taking measurements. Note that the curve for 20 °C was shifted vertically by multiplying 5 for clarity. The Guinier plot analysis of the data at 70 °C based on the criteria of qR_g < 1.3 was shown in the inset, which gives R_g = 3.2 nm. (b) The corresponding WAXS profiles of the PNMG-*b*-PNOG methanol solutions near q = 0.3 measured at 70 °C and 20 °C. Note that all WAXS data were neither converted into an absolute scale, nor subjected to solvent subtraction.

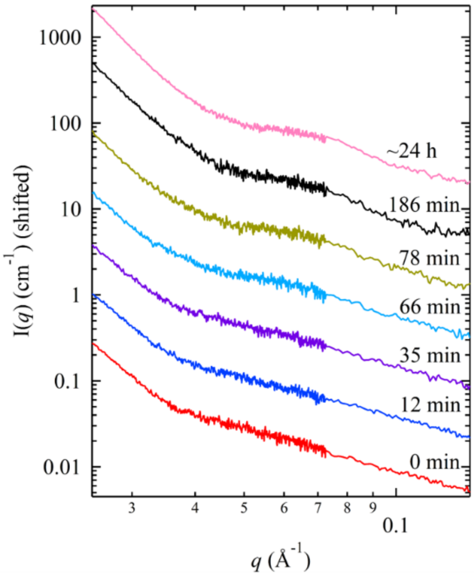

Figure S10. SAXS profiles for the 5 mg/mL PNMG-*b*-PNOG methanol solution at q = 0.025 ~ 0.14 Å^{-1} at different waiting times (t) after been cooled down to room temperature. The profiles were shifted vertically for clarity.

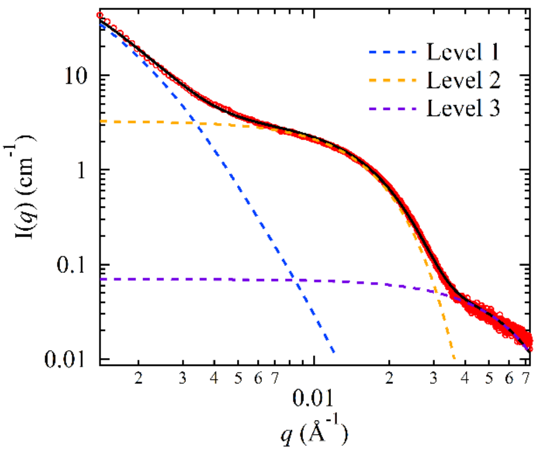

Figure S11. SAXS profiles of the 5 mg/mL PNMG-*b*-PNOG methanol solutions at 0 min after been cooled down to room temperature. A three-level Beaucage unified function[S13] was applied to fit the data, which assumes that the solution is composed of large aggregates of spherical particles (level 1), individual spherical particles (level 2) and uncrystallized polymer coils (level 3) (see above Beaucage Unified Function for details). Note that the R_g of individual polymer coils (level 3) was fixed to 3.2 nm during the fitting based on the high temperature SAXS result (Figure S9). From the best-fit to the data (solid black line), the radius of gyration of spherical particles (*i.e.*, early formed spherical micelles, level 2) is estimated to be 11.5 nm (corresponding to a radius of 14.8 nm via $R_g^2 = 3/5\ R^2$), which is in good agreement with the AFM results (Figure 3a in the main text).

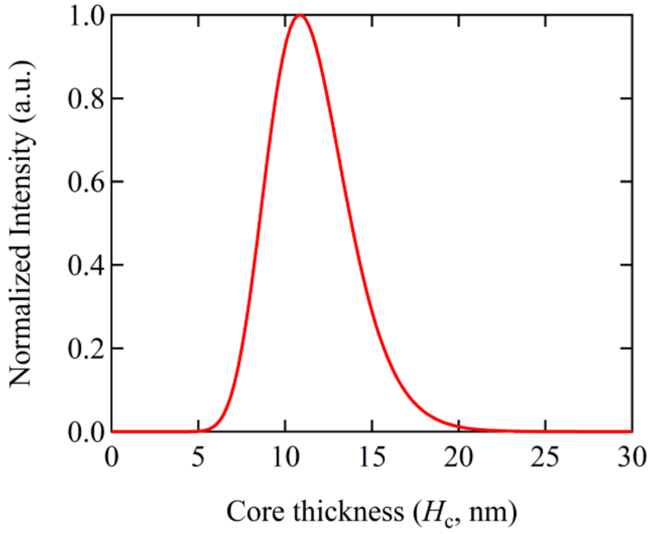

Figure S12. The core thickness (H_c) distribution of PNMG-*b*-PNEHG hexagonal nanosheets in methanol (5 mg/mL) obtained from the best-fit to the SAXS data.

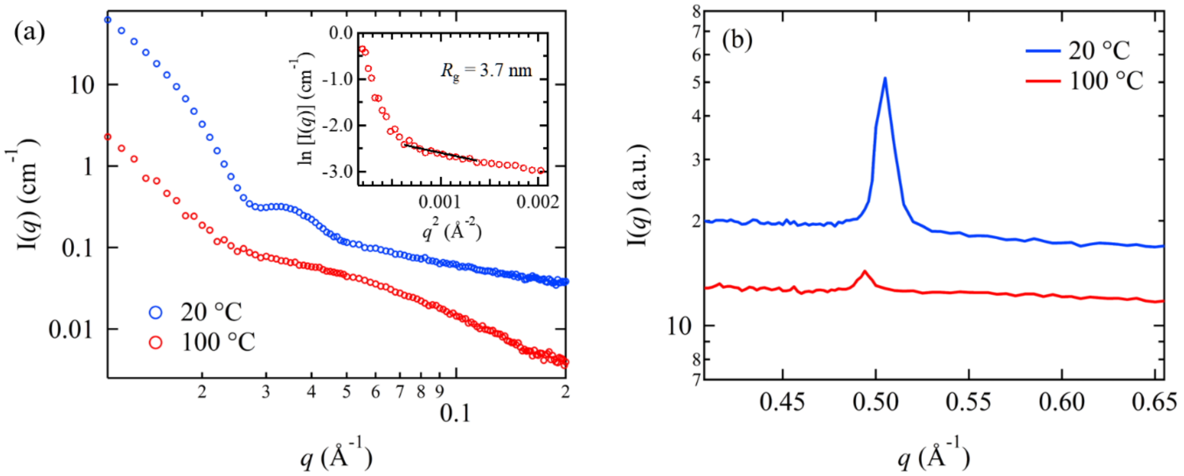

Figure S13. (a) SAXS intensity profiles for the 5 mg/mL PNMG-*b*-PNEHG in methanol measured at 100 °C (red circles) and 20 °C (before heating, blue circles) using a sealed capillary tube. For the measurement took at 100 °C, the sample was first heated and held at 100 °C for 1 min before taking measurements. Note that the curve for 20 °C was shifted vertically by multiplying 10 for clarity. The Guinier plot analysis of the data at 100 °C based on the criteria of $qR_g < 1.3$ was shown in the inset, which gives $R_g = 3.7$ nm. (b) The corresponding WAXS profiles of the PNMG-*b*-PNEHG methanol solutions near $q = 0.5$ measured at 100 °C and 20 °C. Note that all WAXS data were neither converted into an absolute scale, nor subjected to solvent subtraction.

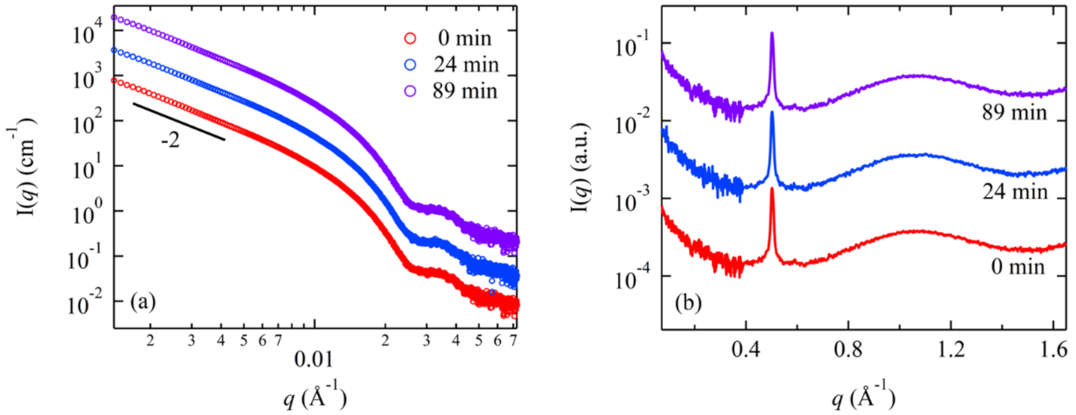

Figure S14. (a) Representative SAXS and (b) WAXS profiles of the 5 mg/mL PNMG-*b*-PNEHG methanol solution at different waiting times (t) after been cooled down to room temperature. The SAXS profiles of 24 and 89 min in (a) were shifted by multiplying 5 and 5^2 for clarity, respectively. The WAXS profiles of 24 and 89 min in (b) were shifted by multiplying 10 and 10^2 for clarity, respectively.

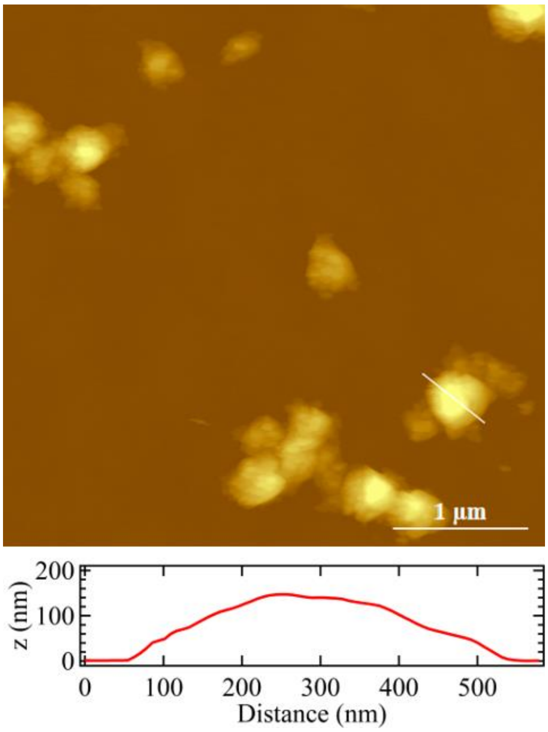

Figure S15. AFM height images for the PNMG-*b*-PNOG self-assembled from methanol solutions with an initial concentration of 10 mg/mL. The height profile along the white line obtained from AFM cross sectional analysis is shown below the image.

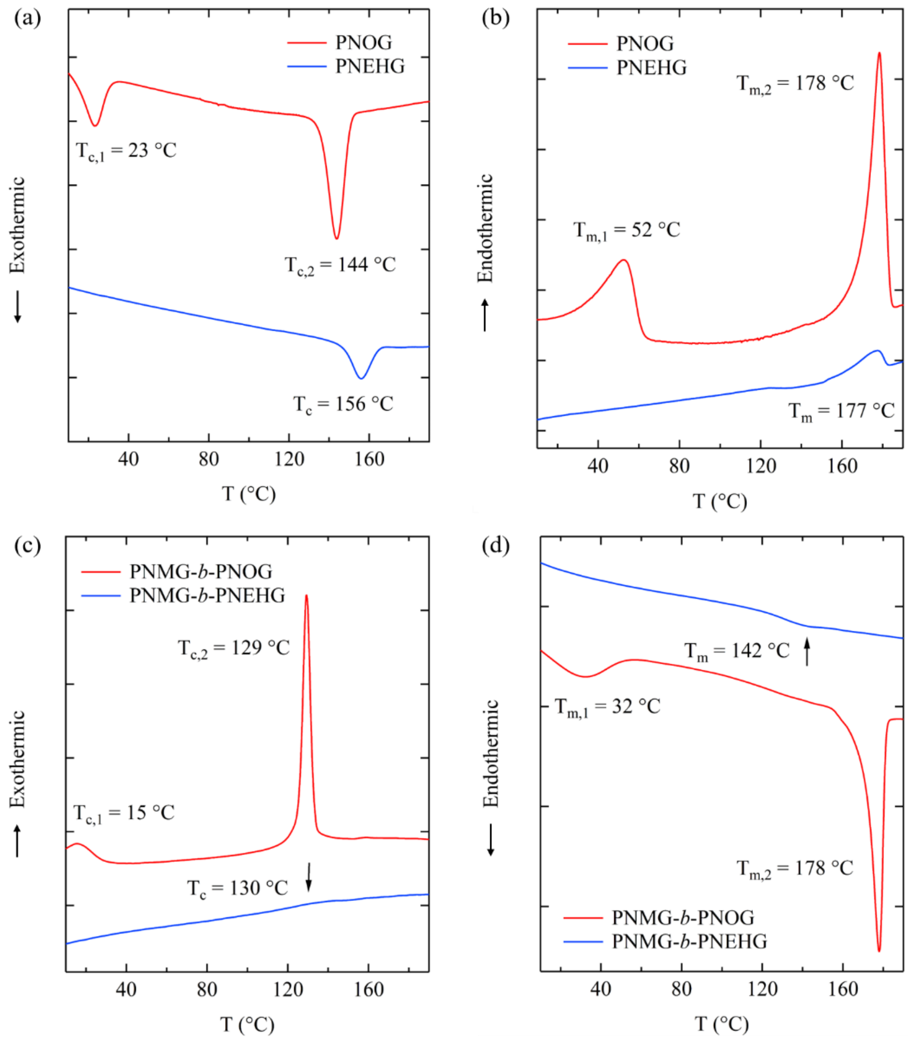

Figure S16. (a-b) DSC thermograms of PNOG (DP_n = 103) and PNEHG (DP_n = 100) homopolymers during (a) first cooling cycle and (b) second heating cycle. (c-d) DSC thermograms of PNMG-*b*-PNOG and PNMG-*b*-PNEHG block copolymers during (c) first cooling cycle and (d) second heating cycle. The PNEHG homopolymer shows a weak exothermic peak (ΔH_c = 3.6 J/g) at T = 156 °C during first cooling as well as a weak endothermic peak (ΔH_m = 4.6 J/g) at T = 177 °C during second heating. For PNMG-*b*-PNEHG, both exothermic and endothermic peaks become less intense, and the transition temperatures were also found to shift to lower temperatures.

References

S1. Lee, C. U.; Smart, T. P.; Guo, L.; Epps, T. H., 3rd; Zhang, D. Synthesis and characterization of amphiphilic cyclic diblock copolypeptoids from *n*-heterocyclic carbene-mediated zwitterionic polymerization of *n*-substituted *n*-carboxyanhydride. *Macromolecules* **2011,** 44, (24), 9574-9585.

S2. Guo, L.; Zhang, D. Cyclic poly(α-peptoid)s and their block copolymers from n-heterocyclic carbene-mediated ring-opening polymerizations of n-substituted n-carboxylanhydrides. *Journal of the American Chemical Society* **2009,** 131, (50), 18072-18074.
S3. Lee, C. U.; Li, A.; Ghale, K.; Zhang, D. Crystallization and melting behaviors of cyclic and linear polypeptoids with alkyl side chains. *Macromolecules* **2013,** 46, (20), 8213-8223.
S4. David, N.; Petr, K. Gwyddion: An open-source software for spm data analysis. *Open Physics* **2012,** 10, (1), 181-188.
S5. Ilavsky, J.; Jemian, P. R. Irena: Tool suite for modeling and analysis of small-angle scattering. *Journal of Applied Crystallography* **2009,** 42, (2), 347-353.
S6. Ilavsky, J. Nika: Software for two-dimensional data reduction. *Journal of Applied Crystallography* **2012,** 45, (2), 324-328.
S7. Pedersen, J. S.; Gerstenberg, M. C. Scattering form factor of block copolymer micelles. *Macromolecules* **1996,** 29, (4), 1363-1365.
S8. Pedersen, J. S. Form factors of block copolymer micelles with spherical, ellipsoidal and cylindrical cores. *Journal of Applied Crystallography* **2000,** 33, (3-1), 637-640.
S9. Sun, J.; Teran, A. A.; Liao, X.; Balsara, N. P.; Zuckermann, R. N. Nanoscale phase separation in sequence-defined peptoid diblock copolymers. *Journal of the American Chemical Society* **2013,** 135, (38), 14119-14124.
S10. Lau, K. H. A.; Ren, C.; Sileika, T. S.; Park, S. H.; Szleifer, I.; Messersmith, P. B. Surface-grafted polysarcosine as a peptoid antifouling polymer brush. *Langmuir* **2012,** 28, (46), 16099-16107.
S11. I. Breßler, J. K. a. A. F. T. *Sasfit*: A tool for small-angle scattering data analysis using a library of analytical expressions. *Journal of Applied Crystallography* **2015,** 48, (5), 1587-1598.
S12. Debye, P. Molecular-weight determination by light scattering. *The Journal of Physical and Colloid Chemistry* **1947,** 51, (1), 18-32.
S13. Beaucage, G.; Schaefer, D. W. Structural studies of complex systems using small-angle scattering: A unified guinier/power-law approach. *Journal of Non-Crystalline Solids* **1994,** 172-174, 797-805.
S14. Beaucage, G.; Kammler, H. K.; Pratsinis, S. E. Particle size distributions from small-angle scattering using global scattering functions. *Journal of Applied Crystallography* **2004,** 37, (4), 523-535.
